# hmmIBD: software to infer pairwise identity by descent between haploid genotypes

**DOI:** 10.1186/s12936-018-2349-7

**Published:** 2018-05-15

**Authors:** Stephen F. Schaffner, Aimee R. Taylor, Wesley Wong, Dyann F. Wirth, Daniel E. Neafsey

**Affiliations:** 1 Infectious Disease and Microbiome Program, Broad Institute of MIT and Harvard, Cambridge, MA 02142 USA; 2 Department of Epidemiology and Center for Communicable Disease Dynamics, Harvard T. H. Chan School of Public Health, Boston, MA 02115 USA; 3 Department of Immunology and Infectious Diseases, Harvard T. H. Chan School of Public Health, Boston, MA 02115 USA; 4 Present Address: Institute for Disease Modeling, Bellevue, WA USA

**Keywords:** Identity by descent, Hidden Markov model, Malaria, Haploid, *Plasmodium falciparum*

## Abstract

**Background:**

A number of recent malaria studies have used identity by descent (IBD) to study epidemiological processes relevant to malaria control. In this paper, a software package, hmmIBD, is introduced for estimating pairwise IBD between haploid genomes, such as those of the malaria parasite, sampled from one or two populations. Source code is freely available.

**Methods:**

The performance of hmmIBD was verified using simulated data and benchmarked against an existing method for detecting IBD within populations. Code for all tests is freely available. The utility of hmmIBD for detecting IBD across populations was demonstrated using *Plasmodium falciparum* data from Cambodia and Ghana.

**Results:**

Alongside an existing method, hmmIBD was highly accurate, sensitive and specific. It is fast, requiring only 70 s on average to analyse 50 whole genome sequences on a laptop computer, and scales linearly in the number of pairwise comparisons. Treatment of different populations under hmmIBD improves detection of IBD across populations.

**Conclusion:**

Fast and accurate software for detecting IBD in malaria parasite genetic data sampled from one or two populations is presented. The latter will likely be a useful feature for malaria elimination efforts, since it could facilitate identification of imported malaria cases. Software is robust to possible misspecification of the genotyping error and the recombination rate. However, exclusion of data in regions whose rates vary greatly from their genome-wide average is recommended.

**Electronic supplementary material:**

The online version of this article (10.1186/s12936-018-2349-7) contains supplementary material, which is available to authorized users.

## Background

Segments of DNA that are shared between individuals and inherited without recombination from a common ancestor are said to be identical by descent (IBD). IBD is a fundamental concept in genetics, linking ancestry to variation due to recombination, which acts on a shorter timescale than mutation [1]. In the field of human genetics, IBD-based analyses are used in many different applications: to map disease loci and quantitative traits, to phase and impute genotypes, and to infer demographic histories [1, 2].

Increasingly, IBD-based analyses are also being used to study haploid organisms such as the malaria parasite. Examples include studies of malaria disease transmission [3], malaria parasites within multiple-genotype infections [4], signals of parasite selection [5], anti-malarial resistance [6], relatedness across proximal parasite populations [7], and the relatedness of co-transmitted parasites strains [8]. However, most existing IBD detection software (recently reviewed in [9]) is designed for humans and other diploids. Accordingly, malaria studies have typically used one of two programs designed specifically for haploids: isoRelate, used and described in [5] and based on [10], and hmmIBD, which was used in [3, 4, 6–8], but has not been fully described in the literature to date.

This paper provides a full description of hmmIBD, its verification using simulated data, and validation and benchmarking of its output and performance against isoRelate. Also described is a novel feature of the program, one that allows inference of IBD between samples from distinct populations.

## Methods

hmmIBD is based on a discrete, heterogeneous, first-order HMM with two hidden states, IBD and not-IBD; mathematical details can be found in Additional file 1. It is designed to infer IBD segments shared between pairs of haploid genomes and to estimate two quantities: (1) the marginal posterior probability of the IBD state (which can be interpreted as the expected fraction of a pair of genomes that is IBD); and (2) the rate at which the genomes transition between states, parameterized by the number of outcrossed meioses since their most recent common ancestor (MRCA), which we refer to as the number of generations. These parameters are estimated iteratively using the Baum-Welch estimation-maximization algorithm; the Viterbi algorithm is then used to calculate the most probable sequence of IBD segments [11].

Model specification requires three probability measures [11]. First, initial state probabilities (IBD or not at the first position on a chromosome). These are initially set to 0.5, and then updated as the expected fraction IBD is recalculated for the entire genome under successive fits of the model. Second, the probabilities of changing state between two genomic positions. These state transition probabilities are functions of the distances between positions (in base pairs), the recombination rate and the number of generations since the MRCA (both assumed to be uniform across the genome), and the expected fraction IBD. Third, emission probabilities, which are the probabilities of the observed allelic types given IBD or not. These are calculated as follows. If two genomes are IBD at a given genomic position, they are of the same allelic type, meaning no mutations are assumed to have occurred since the MRCA. If they are not IBD, the alleles are modelled as independent draws based on the allele frequencies in the population. The probability of the observed alleles is then calculated from these probabilities by allowing for genotyping error.

Parameters include the recombination rate (the default value in the code is for *Plasmodium falciparum* [12]) and the genotyping error rate, as well as estimates of the allele frequencies for all variable sites in the input dataset. Allele frequencies can be supplied by the user; if not supplied, the program estimates them from the data. The model accommodates positions with missing data by omitting emission probabilities at those sites. The program is implemented in C; it complies with the C11 standard and requires no additional libraries. It can accept genotype data for any variant that can be represented by an integer at a single position on the chromosome (e.g. all SNPs and most small indels).

One important assumption of the model is that all IBD regions present between two samples result from common ancestry on a similar timescale. Clearly, this need not be the case: very recent inbreeding can be present along with much older background sharing, the kind that generates linkage disequilibrium in the population. Because IBD deriving from recent common ancestry is of primary interest for many applications, hmmIBD provides an option of capping the number of generations to the MRCA in the model; its effect is to bias against breaking up segments of either state.

## Results

The correctness of hmmIBD’s algorithm and code was verified using data simulated under the HMM on which it is based; details and full results can be found in Additional file 2. For a typical situation, the root-mean-square (RMS) error on the fraction of the genome called as IBD was 0.25 percentage points and on the number of generations was 2 generations. CPU time was linear in the number of variants and quadratic in the number of samples. The performance of hmmIBD was then validated and benchmarked against isoRelate [5] using data created from artificially recombined field samples; details and full results can be found in Additional file 3. Both programs performed well, with accuracies, sensitivities and specificities greater than 98% (Table 1), and performance remained high when genotyping error equal to 0.5% in the artificially recombined genomes was misspecified under the model at 0.1% (Additional file 3). On average, hmmIBD was approximately 24 times faster than isoRelate, although both perform adequately in real time. (We note that isoRelate has a unique capability: by modelling the hidden state space as a set of IBD allele counts in 0, 1 or 2, it can accommodate comparisons across samples containing two genotypes.). The recombination rate under hmmIBD is assumed uniform. Given deviations within a biologically informed range [12], the assumption has little impact upon inference on IBD under hmmIBD using data within the accessible genome.

**Table 1:** Summary of average results based on IBD segments of artificially recombined field data with standard deviations in parentheses; full details can be found in Additional file 3

	Clock time per 50 samples (s)	Accuracy	Sensitivity	Specificity
isoRelate	1774 (372)	0.995 (0.005)	0.999 (0.002)	0.991 (0.008)
hmmIBD	72 (15)	0.992 (0.006)	0.999 (0.001)	0.986 (0.011)

**Fig. 1:**
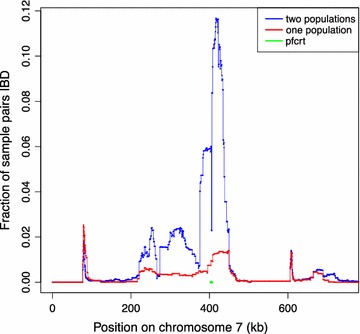
The fraction of sample pairs that are IBD along chromosome 7, where one sample is from Ghana and the other from Cambodia. Blue curve: IBD as reconstructed by hmmIBD correctly treating the samples as coming from two populations; red curve: IBD as reconstructed from a single, averaged population. (See Additional file 2 for details.)

An unusual feature of hmmIBD is that it can accommodate samples from different populations, even ones with very different allele frequencies. This feature should have multiple applications in studying malaria, including detection of selective sweeps that spread between populations and determining the source of imported cases in elimination settings. The effectiveness of hmmIBD can be seen by using it to examine IBD in the region of the well-known selective sweep for chloroquine resistance in *P. falciparum* around the gene *pfcrt* [13]. Figure 1 shows the amount of IBD between field isolates from Ghana and those from Cambodia. The increase in IBD around *pfcrt* is clear, reflecting the fact that the resistance haplotype emerged and spread into Africa from South-East Asia [14, 15]. An alternative approach using hmmIBD but treating the samples as coming from a single population as in [5], also shown, is much less effective at detecting the cross-population IBD.

## Discussion

This study provides a stand-alone description of hmmIBD, software to infer IBD between haploid genomes, and its comparison to isoRelate. Although both programs performed well, they differ in speed (hmmIBD being approximately 24 times faster than isoRelate) and in practical application. Most notably, isoRelate can handle comparisons across samples containing two genotypes where hmmIBD cannot, while hmmIBD can handle comparisons across populations where isoRelate cannot. The latter application is potentially useful for WHO malaria elimination certification, which requires no local cases for at least 3 years, but allows for imported ones [16].

hmmIBD continues to perform well even when the genotyping error and recombination rate is misspecified, and appears to be relatively robust to violation of the assumed uniformity of the recombination rate. These results are unlikely to hold over regions where rates deviate greatly from their average. Exclusion of such regions in data analysed under hmmIBD is therefore recommended. It is also advisable to change the default error rate in the code if a more accurate rate is available. Similarly, if used with a species other than *P. falciparum*, the appropriate average recombination rate should be used. In both cases, specifying an incorrect parameter is likely to affect primarily the number of generations rather than the fraction IBD, providing regions that deviate greatly from the average parameters are excluded. Finally, large public datasets (e.g. from the Pf3k project) may provide better estimates of allele frequencies in a population than can be calculated from the data immediately available to a user; it is advisable to make use of them where available.

## Conclusion

There has been a recent proliferation in the number of malaria studies using IBD. As interest in IBD grows, the need to provide comprehensive details of software used to infer IBD increases. hmmIBD is the only program known to the authors that is designed specifically for haploid malaria genomes and is capable of comparing samples across populations with different allele frequencies. This will likely be a useful feature for malaria elimination efforts, since it could facilitate identification of imported malaria cases.

## Additional files

**Additional file 1.** The hidden Markov model of hmmIBD. Mathematical details of the model behind hmmIBD.

**Additional file 2.** Validation using simulated data; cross-population application. Full details and results of validation and cross-population study.

**Additional file 3.** Comparative study of isoRelate and hmmIBD and impact of assumed uniform recombination under hmmIBD. Full details and results of comparative study and exploration of the impact of assumed uniform recombination under hmmIBD.
