# Supplementary material for: hmmIBD: software to infer pairwise identity by descent between haploid genotypes
Source: Malar J. 2018 May 15;17:196. doi: 10.1186/s12936-018-2349-7 (PMC5952413; doi:10.1186/s12936-018-2349-7)
Supplement: Supplementary file 1 — Additional file 1. The hidden Markov model of hmmIBD. Mathematical details of the model behind hmmIBD. [file 12936_2018_2349_MOESM1_ESM.pdf]

Additional file 1: The hidden Markov model of hmmIBD

April 26, 2018

1 Introduction

In this document we outline a first-order hidden Markov model (HMM) to estimate identity by descent (IBD) between pairs of haploid genotypes from the same or different populations. Notation follows hmmIBD code and that of [2]. Consider a pair of haploid genotypes. At each position along the genome, the pair is considered to be in one of two hidden states, IBD or not IBD. We use genetic data to infer the hidden states. More specifically, for $t = 1, \ldots, T$ genotyped positions, we observe whether the allelic types of the genotype calls of the first and second sample (G_{I_t} and G_{II_t}, respectively) are the same (*homo* if $G_{\mathrm{I}_t} = G_{\mathrm{II}_t}$) or different (*het* if $G_{\mathrm{I}_t} \neq G_{\mathrm{II}_t}$), as illustrated in Table 1, and use the resulting sequence of observations, $\boldsymbol{O} = (O_1, \ldots, O_T)$, to infer the hidden states at the corresponding positions, $\boldsymbol{Q} = (q_1, \ldots, q_T)$. Since the genotype calls may differ from the true underlying genotypes (g_{I_t} and g_{II_t}, respectively), we include an error term, ϵ. The model makes the following assumptions.

1. Conditional on the $t-1$th state, the tth state is independent of the $t-2$th, $\ldots$, 1st states.
2. Conditional on underlying genotypes, genotype calls are independent.
3. Underlying genotypes are dependent given IBD and independent otherwise.
4. The probability of an incorrect genotype call (calling one allelic type as another), ϵ, is constant, such that the probability of a correct genotype call, $(1 - \gamma_t \epsilon)$, decreases with the number of alternative genotypes at the tth position, γ_t.
5. The recombination rate ρ is uniform across and between genomes.
6. Perfect knowledge of genotype frequencies, $\boldsymbol{f}_{g_{\mathrm{I}}} = (f_{g_{\mathrm{I}_1}} \ldots f_{g_{\mathrm{I}_T}})$ and $\boldsymbol{f}_{g_{\mathrm{II}}} = (f_{g_{\mathrm{II}_1}} \ldots f_{g_{\mathrm{II}_T}})$, given frequencies of the observed genotype calls, $\boldsymbol{f}_{G_{\mathrm{I}}} = (f_{G_{\mathrm{I}_1}} \ldots f_{G_{\mathrm{I}_T}})$ and $\boldsymbol{f}_{G_{\mathrm{II}}} = (f_{G_{\mathrm{II}_1}} \ldots f_{G_{\mathrm{II}_T}})$, where $\boldsymbol{f}_{G_{\mathrm{I}}}$ and $\boldsymbol{f}_{G_{\mathrm{II}}}$ are either based on the observed data or supplied by the user.
7. The number of generations separating a pair of haploid genotypes, k, is constant across the genome for a given pair of haploid genotypes, such that all IBD segments in a pair of genomes have experienced the same opportunity for recombination.
8. No mutations have occurred in IBD segments.

9. If two samples come from different populations, the prior over the ancestral population given IBD is uniform.

The model supports pairwise comparisons of samples from the same or different populations. If the two samples come from the same population, let $f_{G_{\mathrm{I}_t}}$ denote the frequency of the observed genotype at the tth position of the first sample across a given pairwise comparison, and $f_{G_{\mathrm{II}_t}}$ that of the second sample. If two samples come from the different populations, let sample one be from the first population with genotype frequency $f^{p1}_{G_{\mathrm{I}_t}}$, and sample two be from the second population with genotype frequency $f^{p2}_{G_{\mathrm{II}_t}}$.

Hypothetical dataset							Model inputs for `sid1:sid2`				
t	`chrom`	`pos`	`sid1`	`sid2`	...	`sidn`	$\boldsymbol{O}$	$\boldsymbol{d}$ (bp)	$\boldsymbol{\gamma}$	$\boldsymbol{f}_{G_{\mathrm{I}}}$	$\boldsymbol{f}_{G_{\mathrm{II}}}$
1	1	353274	A	A	...	A	*homo*	∞	1	$f_A = 0.99$	$f_A = 0.99$
2	1	537427	T	A	...	T	*het*	184153	1	$f_T = 0.49$	$f_A = 0.51$
3	2	217337	T	C	...	G	*het*	∞	3	$f_T = 0.12$	$f_C = 0.43$
$\vdots$	$\vdots$	$\vdots$	$\vdots$	$\vdots$	$\vdots$	$\vdots$	$\vdots$	$\vdots$	$\vdots$	$\vdots$	$\vdots$
T	14	3124991	NA	T	...	C	NA	107242	2	NA	$f_C = 0.96$

Table 1: An example of a hypothetical dataset of n samples from a single population (`sid1` to `sidn`) genotyped at $t = 1, \ldots, T$ positions, and corresponding model inputs for the first sample pair `sid1:sid2`. Columns headings, `chrom` and `pos` refer to chromosome and chromosome position, respectively. Model inputs include the observation sequence $\boldsymbol{O}$, a vector of distances in base pairs (bp) ($\boldsymbol{d}$, where $d_t = \mathrm{pos}_t - \mathrm{pos}_{t-1}$ if $\mathrm{pos}_{t-1} < \mathrm{pos}_t$, otherwise ∞), a vector of alternative genotype counts ($\boldsymbol{\gamma}$, where $\gamma_t = |\mathrm{sid1}_t, \ldots, \mathrm{sidn}_t| - 1$) and vectors of frequencies of genotype calls, $\boldsymbol{f}_{G_{\mathrm{I}}}$ and $\boldsymbol{f}_{G_{\mathrm{II}}}$, corresponding to genotype calls of `sid1` and `sid2` at positions $t = 1, \ldots, T$, respectively.

2 Model specification

The HMM $bm\lambda$ is first-order, discrete, and heterogeneous over $t = 1, \ldots, T$. It is fully specified by the following.

1. $N = 2$ hidden states: $S_1 = 0$ (IBD), and $S_2 = 1$ (not IBD).

2. $M = 2$ observation symbols: $V_1 = \textit{homo}$ (if $G_{\mathrm{I}_t} = G_{\mathrm{II}_t}$) and $V_2 = \textit{het}$ (if $G_{\mathrm{I}_t} \neq G_{\mathrm{II}_t}$). This formulation accommodates observations from within population multiple-genotype samples in which two unphased genotypes reside up to a multiplicative factor of 2.

3. N initial state probabilities, $\boldsymbol{\pi} = (\pi_1, \pi_2)$, where $\pi_i = \mathbb{P}(q_1 = S_i)$ for $i = 1, 2$ and $\sum_{i=1}^{N} (\pi_i) = 1$. These are initially set to 0.5, then updated to the marginal posterior probabilities of the hidden states, $\mathbb{P}(S_1 \mid \boldsymbol{O}, \boldsymbol{\lambda}) = \frac{1}{T} \sum_{t=1}^{T} \mathbb{P}(q_t = S_1 \mid \boldsymbol{O}, \boldsymbol{\lambda})$ and $\mathbb{P}(S_2 \mid \boldsymbol{O}, \boldsymbol{\lambda}) = 1 - \mathbb{P}(S_1 \mid \boldsymbol{O}, \boldsymbol{\lambda})$,

as $\mathbb{P}(S_1 \mid \boldsymbol{O}, \boldsymbol{\lambda})$ is recalculated upon successive fitting iterations of the model, described below (Section 3).

4. An N by N matrix of transition probabilities, $\boldsymbol{A}(t) = \{a_{ij}(t)\}$, where $a_{ij}(t) = \mathbb{P}(q_t = S_j \mid q_{t-1} = S_i)$ for $i, j = 1, 2$ and $\sum_{j=1}^{N} a_{ij}(t) = 1$. Transition probabilities are dependent on the genome position because they vary with the distance in base pairs (bp) of the tth observation from the $t-1$th observation, d_t. They also depend upon π_1 and π_2, the generation number, k, and the recombination rate, ρ (bp^{-1}), which are fixed over $t = 1, \ldots, T$. This formulation allows for the transition probabilities to converge to initial probabilities when d_t, ρ, or k are large,

$$\boldsymbol{A}(t) = \begin{pmatrix} 1 - \pi_2(1 - e^{-k\rho d_t}) & \pi_2(1 - e^{-k\rho d_t}) \\ \pi_1(1 - e^{-k\rho d_t}) & 1 - \pi_1(1 - e^{-k\rho d_t}) \end{pmatrix}. \tag{1}$$

5. An N by M matrix of observation probabilities, $\boldsymbol{B}(t) = \{b_{jk}(t)\}$, where $b_{jk}(t) = \mathbb{P}(O_t = V_k | q_t = S_j)$ for $j, k = 1, 2$,

$$\begin{aligned}
\mathbb{P}(\mathit{homo} \mid \text{IBD}) = b_{11}(t) &= (1 - \gamma_t\epsilon)^2 \bar{f}_{G_t} + \epsilon^2(1 - \bar{f}_{G_t}), & (2)\\
\mathbb{P}(\mathit{homo} \mid \text{not IBD}) = b_{21}(t) &= (1 - \gamma_t\epsilon)^2 f^{p1}_{G_{\mathrm{I}t}} f^{p2}_{G_{\mathrm{II}t}} \\
&\quad + (1 - \gamma_t\epsilon)\epsilon\big(f^{p1}_{G_{\mathrm{I}t}}(1 - f^{p2}_{G_{\mathrm{II}t}}) + f^{p2}_{G_{\mathrm{II}t}}(1 - f^{p1}_{G_{\mathrm{I}t}})\big) \\
&\quad + \epsilon^2(1 - f^{p1}_{G_{\mathrm{I}t}})(1 - f^{p2}_{G_{\mathrm{II}t}}), & (3)\\
\mathbb{P}(\mathit{het} \mid \text{IBD}) = b_{12}(t) &= (1 - \gamma_t\epsilon)\epsilon(\bar{f}_{G_{\mathrm{I}t}} + \bar{f}_{G_{\mathrm{II}t}}) + \epsilon^2(1 - \bar{f}_{G_{\mathrm{I}t}} - \bar{f}_{G_{\mathrm{II}t}}), & (4)\\
\mathbb{P}(\mathit{het} \mid \text{not IBD}) = b_{22}(t) &= (1 - \gamma_t\epsilon)^2 f^{p1}_{G_{\mathrm{I}t}} f^{p2}_{G_{\mathrm{II}t}} \\
&\quad + (1 - \gamma_t\epsilon)\epsilon\big(f^{p1}_{G_{\mathrm{I}t}}(1 - f^{p2}_{G_{\mathrm{II}t}}) + f^{p2}_{G_{\mathrm{II}t}}(1 - f^{p1}_{G_{\mathrm{I}t}})\big) \\
&\quad + \epsilon^2(1 - f^{p1}_{G_{\mathrm{I}t}})(1 - f^{p2}_{G_{\mathrm{II}t}}), & (5)
\end{aligned}$$

where $\bar{f}_{G_t} = \frac{1}{2}(f^{p1}_{G_{\mathrm{I}t}} + f^{p2}_{G_{\mathrm{II}t}})$, $\bar{f}_{G_{\mathrm{I}t}} = \frac{1}{2}(f^{p1}_{G_{\mathrm{I}t}} + f^{p2}_{G_{\mathrm{I}t}})$, and $\bar{f}_{G_{\mathrm{II}t}} = \frac{1}{2}(f^{p1}_{G_{\mathrm{II}t}} + f^{p2}_{G_{\mathrm{II}t}})$. That is to say, we assume a uniform prior over the ancestral population given IBD. Note that when both samples come from the same population ($p1 = p2$), (2) to (5) can be reduced to (6) to (9), since $\bar{f}_{G_t} = f_{G_{\mathrm{I}t}}$ and $f^{p2}_{G_{\mathrm{II}t}} = f^{p1}_{G_{\mathrm{I}t}} = f_{G_{\mathrm{I}t}}$ where $O_t = \mathit{homo}$, while $\bar{f}_{G_{\mathrm{I}t}} = f_{G_{\mathrm{I}t}}$ and $\bar{f}_{G_{\mathrm{II}t}} = f_{G_{\mathrm{II}t}}$, and $f^{p1}_{G_{\mathrm{I}t}} = f_{G_{\mathrm{I}t}}$ and $f^{p2}_{G_{\mathrm{II}t}} = f_{G_{\mathrm{II}t}}$ where $O_t = \mathit{het}$,

$$b_{11}(t) = (1-\gamma_t\epsilon)^2 f_{G_{\mathrm{I}_t}} + \epsilon^2(1-f_{G_{\mathrm{I}_t}}), \tag{6}$$

$$b_{21}(t) = (1-\gamma_t\epsilon)^2 f_{G_{\mathrm{I}_t}}^2 + 2(1-\gamma_t\epsilon)\epsilon f_{G_{\mathrm{I}_t}}(1-f_{G_{\mathrm{I}_t}}) + \epsilon^2(1-f_{G_{\mathrm{I}_t}})^2, \tag{7}$$

$$b_{12}(t) = (1-\gamma_t\epsilon)\epsilon(f_{G_{\mathrm{I}_t}} + f_{G_{\mathrm{II}_t}}) + \epsilon^2(1-f_{G_{\mathrm{I}_t}} - f_{G_{\mathrm{II}_t}}), \tag{8}$$

$$\begin{aligned} b_{22}(t) = {} & (1-\gamma_t\epsilon)^2 f_{G_{\mathrm{I}_t}} f_{G_{\mathrm{II}_t}} + (1-\gamma_t\epsilon)\epsilon(f_{G_{\mathrm{I}_t}}(1-f_{G_{\mathrm{II}_t}}) + f_{G_{\mathrm{II}_t}}(1-f_{G_{\mathrm{I}_t}})) \\ & + \epsilon^2(1-f_{G_{\mathrm{I}_t}})(1-f_{G_{\mathrm{II}_t}}). \end{aligned} \tag{9}$$

The observation probabilities are dependent on the genome position because the alternative genotype count and frequencies vary over the genome. Note that $\sum_{k=1}^{M} b_{ik}(t) \neq 1$ since $\{b_{jk}\}$ encompass all possibilities by allowing $f_{G_{\mathrm{I}_t}}$ and $f_{G_{\mathrm{II}_t}}$ to represent the observed genotype calls (see Section 4 for an example derivation of observation probabilities at a triallelic position for two samples from the same population). This formulation exploits the fact that, regardless of γ_t, at any given position t, only two genotypes can be called for a pair of haploid genotypes, and so given that frequencies must sum to one, only two frequencies, $f_{G_{\mathrm{I}_t}}$ and $f_{G_{\mathrm{II}_t}}$, need to be defined.

The model framework is summarized in Figure 1. Default values in the code of the parameters and bounds are as follows. The recombination rate, $\rho = 7.4 \times 10^{-7}$M bp^{-1}, is that of *Plasmodium falciparum* [1]. The number of generations, k, is inferred under the model (see below), but can be capped by the user. The distances $\boldsymbol{d} = d_1, \ldots, d_T$ are calculated from the data as illustrated in Table 1. We skip positions $<$ 5bp apart to avoid mutations spanning $>$1 bp, while positions on different chromosomes are considered infinitely separated. In the code, the latter is achieved by fitting data for different chromosomes separately under a given iteration of the model (in other words, data from all chromosomes are fit using common values of $\boldsymbol{\pi}$ and k). The genotyping error is specified as $\epsilon = 0.001$. Frequencies $\boldsymbol{f}_{G_{\mathrm{I}}}$ and $\boldsymbol{f}_{G_{\mathrm{II}}}$ and alternative alelle counts $\boldsymbol{\gamma}$ are either based on input data or on external information provided by the user. To accommodate indels, the maximum value of γ_t is assumed to be 8 but, as with all other specified values, can be changed within the code. In is important to note that all genotype calls, including indels, are treated as point mutations under the model.

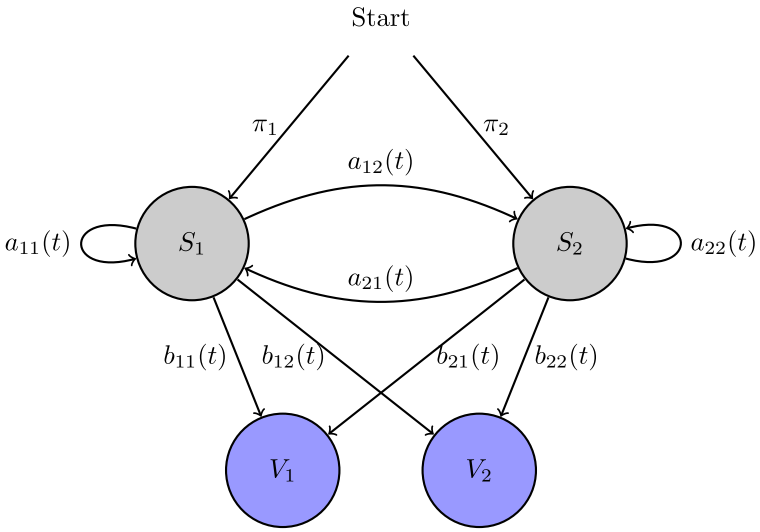

Figure 1: Summary of the HMM framework. Hidden states (S_1 and S_2) and observation symbols (V_1 and V_2) are shown in grey and blue, respectively. Edges are annotated by their respective probability measures.

3 Model implementation

Given the model, $\boldsymbol{\lambda} = (\boldsymbol{\pi}, \boldsymbol{A}, \boldsymbol{B})$ (noting that $\boldsymbol{A}$ is conditional on k), and across all chromosomes a sequence of observations ($\boldsymbol{O} = O_1, \ldots, O_T$), distances ($\boldsymbol{d}$), frequencies ($\boldsymbol{f}_{G_{\mathrm{I}}}$ and $\boldsymbol{f}_{G_{\mathrm{II}}}$), and alternative genotype counts ($\boldsymbol{\gamma}$), we the fit the model as follows.

1. We initialise the model, $\boldsymbol{\lambda}^{[0]}$, setting $k^{[0]} = 1$ and $\boldsymbol{\pi}^{[0]} = (0.5, 0.5)$.
2. Over a given number of fitting iterations, $\tau = 1, \ldots, \tau_{\max}$, which is capped at a user-settable maximum $\tau_{\max}$ that defaults to 5, we update $\boldsymbol{\lambda}$ via parameters k and $\boldsymbol{\pi}$ using the Baum-Welch method [2]. Note that the fitting iterations will stop before the cap if the default convergence criteria are met. Specifically, while $\tau \leq \tau_{max}$ or default convergence criteria are not met,

$$k^{[\tau]} = k^{[\tau-1]} \frac{\sum_t^T \left\{ \frac{\mathbb{P}(q_t = S_1, q_{t+1} = S_2, \boldsymbol{O} \mid \boldsymbol{\lambda}^{[\tau-1]})}{\mathbb{P}(\boldsymbol{O} \mid \boldsymbol{\lambda}^{[\tau-1]})} + \frac{\mathbb{P}(q_t = S_2, q_{t+1} = S_1, \boldsymbol{O} \mid \boldsymbol{\lambda}^{[\tau-1]})}{\mathbb{P}(\boldsymbol{O} \mid \boldsymbol{\lambda}^{[\tau-1]})} \right\}}{\sum_t^T \left\{ \mathbb{P}(q_t = S_1 \mid \boldsymbol{\lambda}^{[\tau-1]}, \boldsymbol{O}) \mathbb{P}(q_{t+1} = S_2 \mid q_t = S_1, \boldsymbol{\lambda}^{[\tau-1]}) + \mathbb{P}(q_t = S_2 \mid \boldsymbol{\lambda}^{[\tau-1]}, \boldsymbol{O}) \mathbb{P}(q_{t+1} = S_1 \mid q_t = S_2, \boldsymbol{\lambda}^{[\tau-1]}) \right\}},$$

$$\boldsymbol{\pi}^{[\tau]} = (\pi_1^{[\tau]}, 1 - \pi_1^{[\tau]}) \text{ where } \pi_1^{[\tau]} = \frac{1}{T} \sum_t^T \mathbb{P}(q_t = S_1 \mid \boldsymbol{O}, \boldsymbol{\lambda}^{[\tau-1]}).$$

3. Having fit $\boldsymbol{\pi}$ and k, we retrieve the most probable sequence of hidden states, argmax $\mathbb{P}(\boldsymbol{Q} \mid \boldsymbol{O}, \boldsymbol{\lambda})$, using the Viterbi algorithm [2].

Observations with one or more missing genotype calls are considered missing (NA in Table 1). Missing observations are easily accounted for within the HMM framework by simply omitting the respective observation probability terms in likelihood calculations. In the code, this is achieved by setting $\mathbb{P}(O_t = \text{NA}|q_t = S_i) = 1 \; \forall \; i = 1, 2$. Practical details regarding implementation, for example how to compile the code, can be found in the corresponding ReadMe file at https://github.com/glipsnort/hmmIBD/releases.

4 Example derivation of observation probabilities for within population samples

Consider a triallelic position where $\lambda_t = 2$. Here we show how to derive the observation probabilities equations (6) to (9). For brevity, we henceforth drop the subscript t. Let the set of three possible genotype calls at the triallelic position be denoted by $\{A, B, C\}$, and the equivalent set of genotypes be denoted by $\{a, b, c\}$. The probability of a genotype call, $G \in \{A, B, C\}$, given an underlying genotype, $g \in \{a, b, c\}$, is given by,

$$\mathbb{P}(G \mid g) = \begin{array}{c} \\ A \\ B \\ C \end{array} \overset{\begin{array}{ccc} a \qquad & \qquad b \qquad & \qquad c \end{array}}{\begin{pmatrix} (1-2\epsilon) & \epsilon & \epsilon \\ \epsilon & (1-2\epsilon) & \epsilon \\ \epsilon & \epsilon & (1-2\epsilon) \end{pmatrix}}, \tag{10}$$

Now let us consider a pair of genotype calls, $(G_{\text{I}}G_{\text{II}})$ where $G_{\text{I}} \in \{A, B, C\}$ and $G_{\text{II}} \in \{A, B, C\}$, and genotypes, $(g_{\text{I}}g_{\text{II}})$ where $g_{\text{I}} \in \{a, b, c\}$ and $g_{\text{II}} \in \{a, b, c\}$. The probabilities of the genotype pairs given the hidden states, $q \in \{S_1, S_2\}$, are

$$\mathbb{P}(g_{\text{I}}g_{\text{II}} \mid q) = \begin{array}{c} \\ aa \\ bb \\ cc \\ ab \\ ac \\ bc \\ ba \\ ca \\ cb \end{array} \overset{\begin{array}{cc} S_1 & S_2 \end{array}}{\begin{pmatrix} f_a & f_a^2 \\ f_b & f_b^2 \\ f_c & f_c^2 \\ 0 & f_a f_b \\ 0 & f_a f_c \\ 0 & f_b f_c \\ 0 & f_a f_b \\ 0 & f_a f_c \\ 0 & f_b f_c \end{pmatrix}}, \tag{11}$$

where f_g is the frequency of a given genotype, due to assumed dependence between genotypes given IBD and independence given not IBD. And, since we are assuming conditional independence between genotype calls given underlying genotypes such that

$$\mathbb{P}\big(G_{\mathrm{I}}G_{\mathrm{II}} \mid q\big) = \sum_{g_{\mathrm{I}}g_{\mathrm{II}}} \mathbb{P}(G_{\mathrm{I}} \mid g_{\mathrm{I}})\mathbb{P}(G_{\mathrm{II}} \mid g_{\mathrm{II}})\mathbb{P}\big(g_{\mathrm{I}}, g_{\mathrm{II}} \mid q\big),$$

where $\sum_{g_{\mathrm{I}},g_{\mathrm{II}}}$ denotes the summation over all possible genotype pairs, the probabilities of all possible homogeneous and heterogeneous comparisons of genotype calls given the hidden states are,

$$\begin{aligned}
\mathbb{P}(AA \mid S_1) &= (1-2\epsilon)^2 f_a + \epsilon^2(f_b + f_c), \\
&= (1-2\epsilon)^2 f_a + \epsilon^2(1 - f_a); && (12)\\
\mathbb{P}(BB \mid S_1) &= (1-2\epsilon)^2 f_b + \epsilon^2(1 - f_b); && (13)\\
\mathbb{P}(CC \mid S_1) &= (1-2\epsilon)^2 f_c + \epsilon^2(1 - f_c); && (14)
\end{aligned}$$

$$\begin{aligned}
\mathbb{P}(AA \mid S_2) &= (1-2\epsilon)^2 f_a^2 + 2(1-2\epsilon)\epsilon(f_a f_b + f_a f_c) + \epsilon^2(f_b f_c + f_b^2 + f_c^2), \\
&= (1-2\epsilon)^2 f_a^2 + 2(1-2\epsilon)\epsilon(f_a(1-f_a)) + \epsilon^2(1-f_a)^2; && (15)\\
\mathbb{P}(BB \mid S_2) &= (1-2\epsilon)^2 f_b^2 + 2(1-2\epsilon)\epsilon(f_b(1-f_b)) + \epsilon^2(1-f_b)^2; && (16)\\
\mathbb{P}(CC \mid S_2) &= (1-2\epsilon)^2 f_c^2 + 2(1-2\epsilon)\epsilon(f_c(1-f_c)) + \epsilon^2(1-f_c)^2; && (17)
\end{aligned}$$

$$\begin{aligned}
\mathbb{P}(AB \mid S_1) &= (1-2\epsilon)\epsilon(f_a + f_b) + \epsilon^2(f_c), \\
&= (1-2\epsilon)\epsilon(f_a + f_b) + \epsilon^2(1 - f_a - f_b); && (18)\\
\mathbb{P}(AC \mid S_1) &= (1-2\epsilon)\epsilon(f_a + f_c) + \epsilon^2(1 - f_a - f_c); && (19)\\
\mathbb{P}(BC \mid S_1) &= (1-2\epsilon)\epsilon(f_b + f_c) + \epsilon^2(1 - f_b - f_c); && (20)\\
\mathbb{P}(BA \mid S_1) &= \mathbb{P}(AB \mid S_1); && (21)\\
\mathbb{P}(CA \mid S_1) &= \mathbb{P}(AC \mid S_1); && (22)\\
\mathbb{P}(CB \mid S_1) &= \mathbb{P}(BC \mid S_1): && (23)
\end{aligned}$$

$$\begin{aligned}
\mathbb{P}(AB \mid S_2) &= (1-2\epsilon)^2 f_a f_b + (1-2\epsilon)\epsilon(f_a(f_a + f_c) + f_b(f_b + f_c)) + \epsilon^2(f_a f_b + f_a f_c + f_b f_c + f_c^2), \\
&= (1-2\epsilon)^2 f_a f_b + (1-2\epsilon)\epsilon(f_a(1-f_b) + f_b(1-f_a)) + \epsilon^2(1 - f_a - f_b + f_a f_b); && (24)\\
\mathbb{P}(AC \mid S_2) &= (1-2\epsilon)^2 f_a f_c + (1-2\epsilon)\epsilon(f_a(1-f_c) + f_c(1-f_a)) + \epsilon^2(1 - f_a - f_c + f_a f_c); && (25)\\
\mathbb{P}(BC \mid S_2) &= 2(1-2\epsilon)^2 f_b f_c + 2(1-2\epsilon)\epsilon(f_b(1-f_c) + f_c(1-f_b)) + 2\epsilon^2(1 - f_b - f_c + f_b f_c); && (26)\\
\mathbb{P}(BA \mid S_2) &= \mathbb{P}(AB \mid S_2); && (27)\\
\mathbb{P}(CA \mid S_2) &= \mathbb{P}(AC \mid S_2); && (28)\\
\mathbb{P}(CB \mid S_2) &= \mathbb{P}(BC \mid S_2); && (29)
\end{aligned}$$

where summations over (12) to (14) and (18) to (23) = 1, and over (15) to (17) and (24) to (29) = 1.

Using $f_{G_{\mathrm{I}}} = f_{G_{\mathrm{II}}}$ to approximate $f_{g_{\mathrm{I}}} = f_{g_{\mathrm{II}}}$, where $f_{G_{\mathrm{I}}} = f_{G_{\mathrm{II}}}$ is the frequency of the genotype call in a homogeneous comparison, and $f_{G_{\mathrm{I}}}$ and $f_{G_{\mathrm{II}}}$ to approximate $f_{g_{\mathrm{I}}}$ and $f_{g_{\mathrm{II}}}$, respectively, where

$f_{G_{\mathrm{I}}}$ and $f_{G_{\mathrm{II}}}$ are the frequencies of the genotype calls in the first and second samples, respectively, in a heterogeneous comparison, equations (12) to (14) can be reduced to equation (6), equations (15) to (17) to equation (7), equations (18) to (23) to equation (8) and equations (24) to (29) to equation (9).

References

[1] A. Miles, Z. Iqbal, P. Vauterin, R. Pearson, S. Campino, M. Theron, K. Gould, D. Mead, E. Drury, J. O. Brien, V. R. Rubio, B. Macinnis, J. Mwangi, U. Samarakoon, L. Ranford-cartwright, M. Ferdig, K. Hayton, X.-z. Su, T. Wellems, J. Rayner, G. Mcvean, and D. Kwiatkowski. Indels, structural variation and recombination drive genomic diversity in Plasmodium falciparum. *Genome Research*, 26(9):1288–1299, 2016.

[2] L. R. Rabiner. A tutorial on hidden Markov models and selected applications in speech recognition. *Proceedings of the IEEE*, 77(2):257–286, 1989.
