# Supplementary material for: hmmIBD: software to infer pairwise identity by descent between haploid genotypes
Source: Malar J. 2018 May 15;17:196. doi: 10.1186/s12936-018-2349-7 (PMC5952413; doi:10.1186/s12936-018-2349-7)
Supplement: Supplementary file 2 — Additional file 2. Validation using simulated data; cross-population application. Full details and results of validation and cross-population study. [file 12936_2018_2349_MOESM2_ESM.docx]

**Additional File 2: Validation using simulated data; cross-population application**

**Simulations**

We validated hmmIBD and evaluated its performance by using it to reconstruct data simulated under the same underlying model; see Appendix S1 for full mathematical details of the model.

Programs, scripts and intermediate data files needed to repeat this analysis can be found in https://github.com/artaylor85/hmmIBD_ benchmark/appendix2; see the readme files in sim and realdata subdirectories.

The simulated samples consisted of complete *P. falciparum* genomes. Variant spacing and frequencies were taken directly from empirical data, specifically the set of Pf3k samples from Ghana (The Pf3K Project (2016): pilot data release 5.0. [www.malariagen.net/data/pf3k-5)](http://www.malariagen.net/data/pf3k-5)). We restricted the samples to those with a high probability of being monogenomic, as determined by DEploid (Zhu, 2017), and filtered variants in that set to include only single nucleotide polymorphisms (SNPs) with at least two copies of the minor allele in the entire Pf3K dataset, and located within the accessible genome (as defined in (Manske, et al., 2012)). SNPs with more than two alleles were treated as bi-allelic for this study, with all non-reference alleles treated as a single allele.

For each simulated genome, an expected fraction IBD and not IBD **π** = (π_1_, π_2_) and a number of generations, k, was chosen. The initial state (IBD or not) for each chromosome was assigned based on **π**. The simulation then iterated through the variants on the chromosomes, randomly assigning genotype calls based on the emission probabilities **B** and making random state changes between variant sites with transition probabilities **A**. The resulting simulated data were then processed with hmmIBD, and fitted parameters compared with the values used to generate the data. Figure S2.1 shows the accuracy of hmmIBD in reconstructing the fraction of the genome that is IBD. S2.2 and S2.3 show the improvement of the output estimates with iterations of the fit. Note that the fit will stop before the maximum number of iterations when the (default) convergence criteria are met.

We similarly validated IBD inference for two populations. In this case, we used variant positions and allele frequencies from the Pf3k Senegal and Thailand samples, filtered as described above. Results are shown in Figures S2.4 and S2.5.

To explore how the number of variants affects the IBD estimates, we prepared simulated datasets as described above, and then thinned them to different degrees (keeping every other marker, every third marker, etc.) before processing with hmmIBD (Figure S2.6, S2.7). Run time was linear in the number of variants used (Figure S2.8) and linear in the number of pairwise comparisons (data not shown).

Finally, we evaluated the effect that uncertainty in allele frequencies had on inference of IBD. To do this, we again started with a genome-wide set of measured allele frequencies from a set of *P. falciparum* samples,

Using these “true” allele frequencies, we generated simulated datasets as described above. We then applied hmmIBD to the simulated data (maximum fit iteration = 40), using re-estimated allele frequencies. The re-estimated frequencies were generated for each SNP by drawing a random sample of size n from a binomial with probability equal to the “true” frequency. Figure S2.9 shows the effect on IBD reconstruction of estimating frequencies with small datasets.

**Cross population results**

To assess the effectiveness of cross-population IBD inference, we compared two approaches to identifying IBD in the region around *pfcrt* on chromosome 7, the location of a drug resistance selective sweep. For this study we used the Ghana and Cambodia Pf3K data, filtered as described above, but without collapsing SNPs with more than 2 alleles. In one approach, we simply applied hmmIBD to the data from the two populations. In the other, adopted from a recent study (Henden, 2016), we filtered out all SNPs with allele frequencies that differed by more 0.30 between the two population samples, and otherwise treated the two populations as one, with a single, averaged allele frequency for each SNP. We applied hmmIBD to this dataset in single-population mode.

For both approaches, we tabulated IBD segments, filtering out those with <100 SNPs and those < 2 kb in length. The results are shown in Figure 1.

Figure S2.1. Reconstructed vs generated IBD fraction. Simulation for *k* = 10 generations.

Figure S2.2. Root-mean-square error on IBD fraction. The simulated IBD fraction is in the range 0.3 ≤ π_1_ ≤ 0.7.

Figure S2.3. RMS error on the number of generations (*k*). Simulated IBD fraction is in the range 0.3 ≤ π_1_ ≤ 0.7.

Figure S2.4. Root-mean-square error on IBD fraction in cross-population comparisons. The simulated IBD fraction is in the range 0.3 ≤ π_1_ ≤ 0.7.

Figure S2.5. RMS error on the number of generations in cross-population comparisons. Simulated IBD fraction is in the range 0.3 ≤ π_1_ ≤ 0.7.

Figure S2.6. Effect of number of variants on IBD estimate. Simulated IBD fraction = 0.5; number of generations = 10.

Figure S2.7. Accuracy of reconstructed IBD fraction using 1% of variants (=1429 variants). Number of generations = 10.

Figure S2.8. Total run time as a function of number of markers (run on a MacBook Pro, 2 GHz Intel Core i7 processor). Simulated IBD fraction = 0.5; number of generations = 10.

Figure S2.9. Effect of error in estimated allele frequency on IBD reconstruction. RMS error in the reconstructed IBD fraction is shown as a function of the sample size used to estimate allele frequencies in the sample set. Simulated IBD fraction = 0.5; number of generations = 10.

**References**

Henden, L.L., S; Mueller, I; Barry, A; Bahlo, M. Detecting Selection Signals In Plasmodium falciparum Using Identity-by-Descent Analysis. *BioArxiv* 2016.

Manske, M.*, et al.* Analysis of Plasmodium falciparum diversity in natural infections by deep sequencing. *Nature* 2012;487(7407):375-379.

Zhu, S.J.A.-G., J; McVean, G. Deconvolution of multiple infections in Plasmodium falciparum from high throughput sequencing data. *BioArxiv* 2017.
