# Supplementary material for: hmmIBD: software to infer pairwise identity by descent between haploid genotypes
Source: Malar J. 2018 May 15;17:196. doi: 10.1186/s12936-018-2349-7 (PMC5952413; doi:10.1186/s12936-018-2349-7)
Supplement: Supplementary file 3 — Additional file 3. Comparative study of isoRelate and hmmIBD and impact of assumed uniform recombination under hmmIBD. Full details and results of comparative study and exploration of the impact of assumed uniform recombination under hmmIBD. [file 12936_2018_2349_MOESM3_ESM.pdf]

Additional file 3: Comparative study of isoRelate and hmmIBD and impact of assumed uniform recombination under hmmIBD

Comparative study of isoRelate and hmmIBD

Methods

Summary of files and variables used

The following section summarizes results generated from a comparative study of hmmIBD and isoRelate (Henden L, et al. BioRxiv. 2016). Analyses were based on data generated by artificial recombination (details below). The steps, data and scripts required to reproduce this study are as follows.

1. Download the hmmIBD_benchmark repository from https://github.com/artaylor85/hmmIBD_benchmark and unzip the pf3k_data directory.
2. Install hmmIBD following instructions at https://github.com/glipsnort/hmmIBD/releases (v2.0.0).
3. Install isoRelate following instructions at https://github.com/bahlolab/isoRelate/releases (results here based on v0.1.0 installed Aug 9th 2017).
4. Set working directory to this source file location.
5. Run `Simulate_chimeric_genotypes.R`.
6. Run `Run_isolate_hmmIBD.R`.
7. Run `Post_process_results.R`.
8. Run/knit the Rmd file that generates Additional_file_3.pdf (i.e. this file).

Once downloaded, the code in `hmmIBD_benchmark` can be modified in any way (e.g. to explore data from sites with fewer than 100 isolates, edit `min_num = 100` in `Simulate_chimeric_genotypes.R`).

Simulation of artificially recombined data

We used artificially recombined data to compare results generated under hmmIBD and isoRelate to a known truth that was not generated under either model. Artificially recombined data were based on the MalariaGen Pf3k samples, pilot release 5.0 (https://www.malariagen.net/projects/pf3k). These data were filtered prior to their use in this comparative study, leaving only single nucleotide polymorphisms (SNPs) in the accessible genome (as defined by Miles A, et al. Genome Research. 2016), and those with a high probability of being monogenomic (as defined by DEploid from Zhu SJ, Almagro-garcia J, Mcvean G. BioRxiv. 2017). The filtered data can be found in `pf3k_data`. Using `Simulate_chimeric_genotypes.R` we:

1. Extracted samples from sites with 100 or more samples (Thies, Kassena, Pursat).
2. For each site, removed multiallelic SNPs (unsupported by isoRelate) and those with minor allele frequency ≤ 0.01, leaving 57307, 41992, 69438 SNPs per sample from Kassena, Pursat, Thies, respectively.
3. Calculated and saved allele frequencies and data sets based on the unrecombined data to ensure frequencies were not based on chimeric samples.
4. For each pairwise comparison within a site, calculated the average identity-by-state, IBS (one minus the genome-wide average SNP difference), and plotted.
5. Extracted unrelated pairs (those with IBS < 1 percentile of the empirical IBS distribution).
6. Artificially recombined each unrelated sample pair to create a "chimeric child". Recombination was simulated by sampling crossover positions (in base pairs, bp) from an exponential distribution with mean equal to the recombination rate in Morgans, M, per bp (see `functions.R`).

7. Recorded the parent of each DNA segment in each chimeric child, and plotted the number of crossovers per chromosome averaged over all the chimeric children per site.

In addition to the above steps, we generated an erroneous copy of each parent and chimeric child. More specifically, for each SNP with probability equal to the genotyping error 0.005, the copied allele was replaced by its biallelic counterpart.

Experiments to evaluate timing

Timing experiments were performed on the first 50 samples per site (including unrecombined parents and non-erroneous chimeric children), and repeated 3 times on a MacBook Air laptop with 1.7 GHz Intel Core i7 processor using the parameter values listed in the table below. Some of the parameter values differ to the defaults provided in order to more closely match the two methods.

Table 1: Specified parameter values. NA denotes not applicable. †In isoRelate, the “recombination rate” is a function of distance in M. The equivalent fixed rate in M/bp in hmmIBD was thus based on the empirical relationship between positions in bp and centimorgans provided in the png_pedmap data set of the isoRelate package.

Parameter	isoRelate	hmmIBD
genotyping error	0.005	0.001
recombination rate	5.83e-07 M/bp†	5.83e-07 M/bp
minimum no. SNPs per segment	0	NA
minimum length (bp) per segment	0	NA
Minimum marker spacing (bp)	NA	0
Minimum informative sites per genome	NA	0

Experiments to evaluate inference

For each site, IBD segments between 50 “chimeric children” and each of their two parents were inferred under isoRelate and hmmIBD using the parameter values listed in the table above. Accuracy, sensitivity and specificity were calculated as follows, where for a given pairwise comparison and SNP, a true positive is an IBD observation given an IBD state, and a true negative is a not IBD (nIBD) observation given a nIBD state,

$$\text{Accuracy} = \frac{\sum \text{ True positive} + \sum \text{ True negative}}{\text{Number of SNPs}}, \tag{1}$$

$$\text{Sensitivity} = \frac{\sum \text{ True positive}}{\sum \text{ IBD states}}, \tag{2}$$

$$\text{Specificity} = \frac{\sum \text{ True negative}}{\sum \text{ nIBD states}}. \tag{3}$$

We also compared estimates of the numbers of generations inferred under isoRelate and hmmIBD, and the proportion simulated IBD with the posterior probability of IBD inferred under hmmIBD (the latter was not directly available under v0.1.0 of isoRelate).

To investigate the impact of genotyping error, the entire process was then repeated for the erroneous copies of 50 chimeric children and their parents. We expect error to introduce small and incorrectly inferred segments into otherwise correctly inferred segments of DNA that are both IBD and not. Concomitantly, we expect error to spuriously increase estimates of numbers of generations since most recent common ancestors.

Results

Timing

Table 2: Clocktime (sec) per 50 samples

	isoRelate	hmmIBD
Kassena 1	1647.566	68.556
Pursat 1	1242.745	45.657
Thies 1	2035.602	74.431
Kassena 2	1648.765	68.567
Pursat 2	1243.346	45.347
Thies 2	2033.767	74.642
Kassena 3	1645.554	68.498
Pursat 3	1242.854	45.473
Thies 3	2037.765	74.799

Table 3: CPU time (sec) per 50 samples

	isoRelate	hmmIBD
Kassena 1	1591.945	68.148
Pursat 1	1196.766	45.365
Thies 1	1960.100	73.969
Kassena 2	1592.889	68.024
Pursat 2	1196.816	45.200
Thies 2	1959.130	74.153
Kassena 3	1590.386	68.154
Pursat 3	1197.044	45.321
Thies 3	1963.492	74.262

Inference based on non-erroneous chimeric children

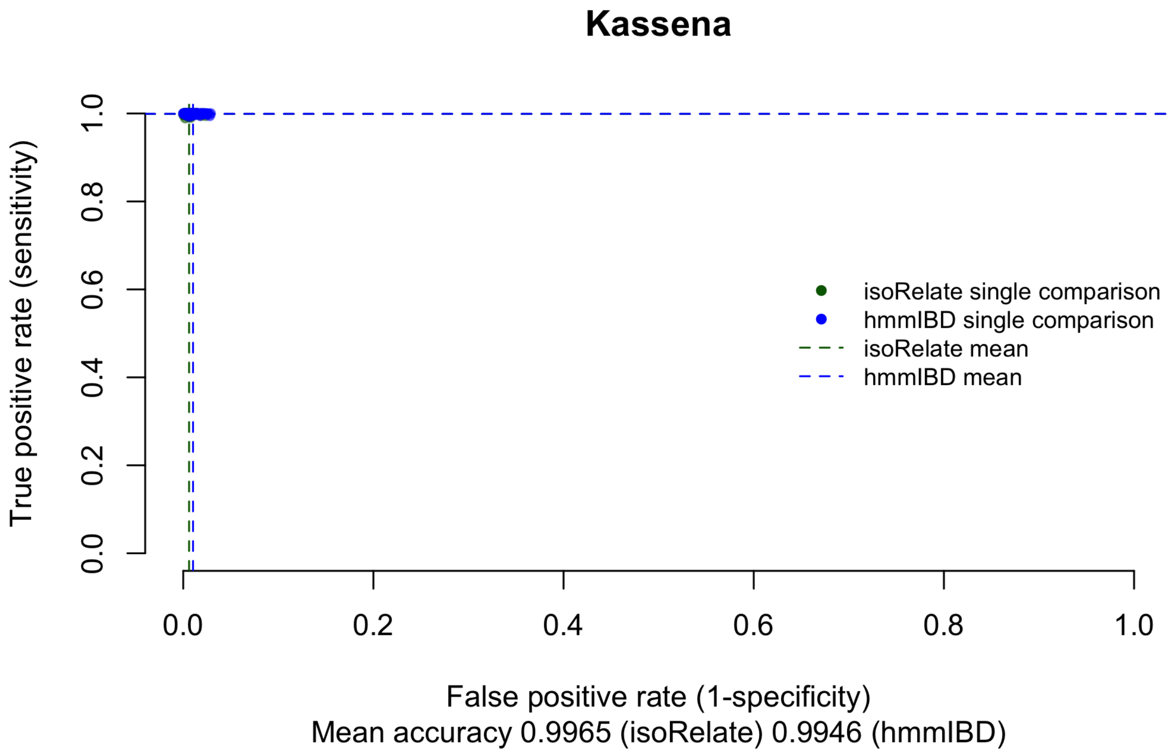

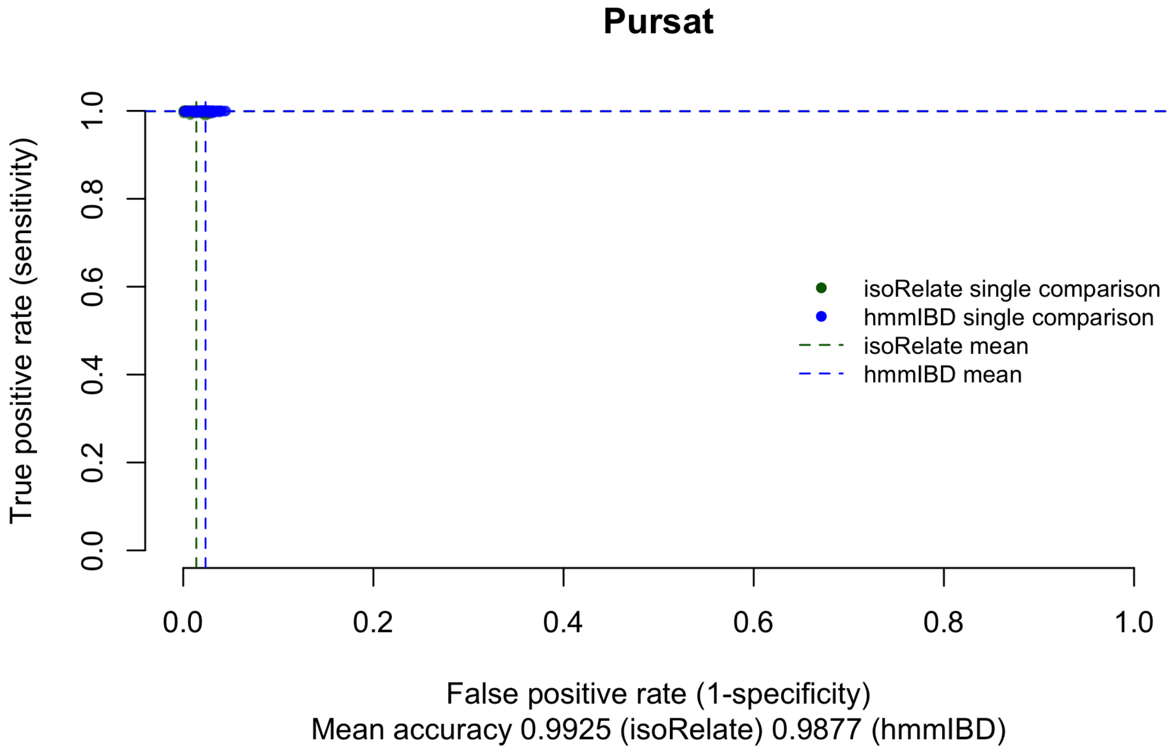

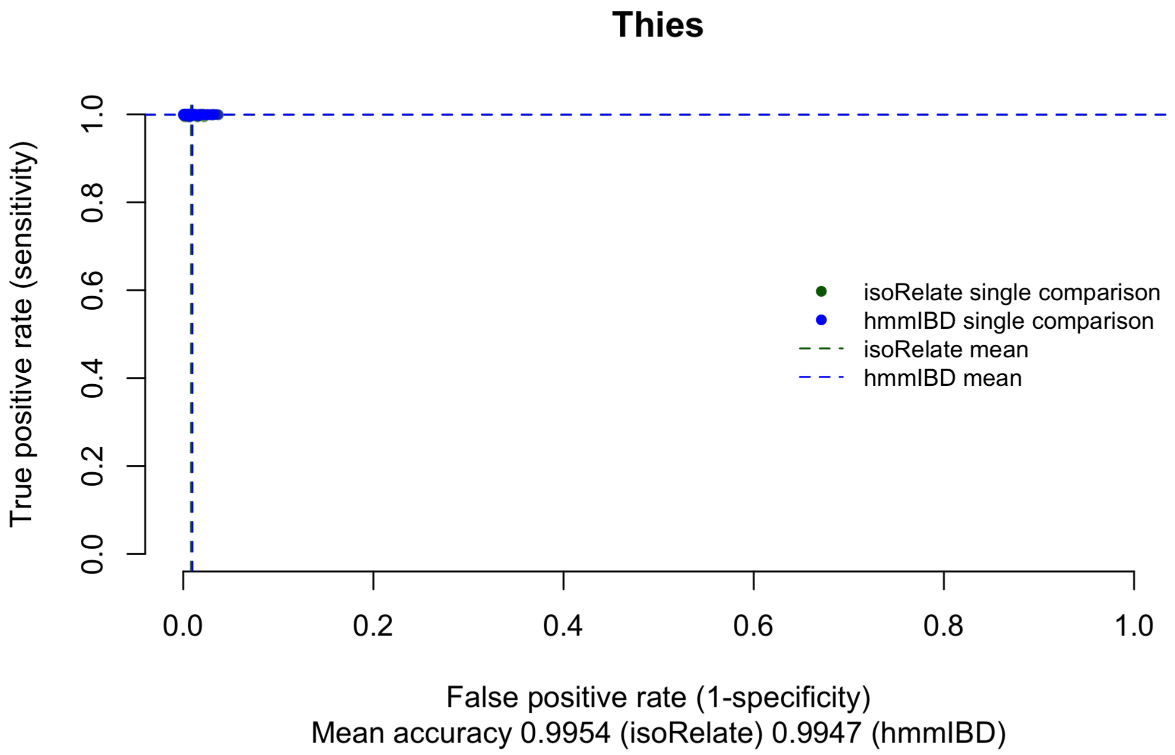

Illustrative assignment plots for two randomly selected pairwise comparisons based on non-erroneous chimeric children from Kassena.

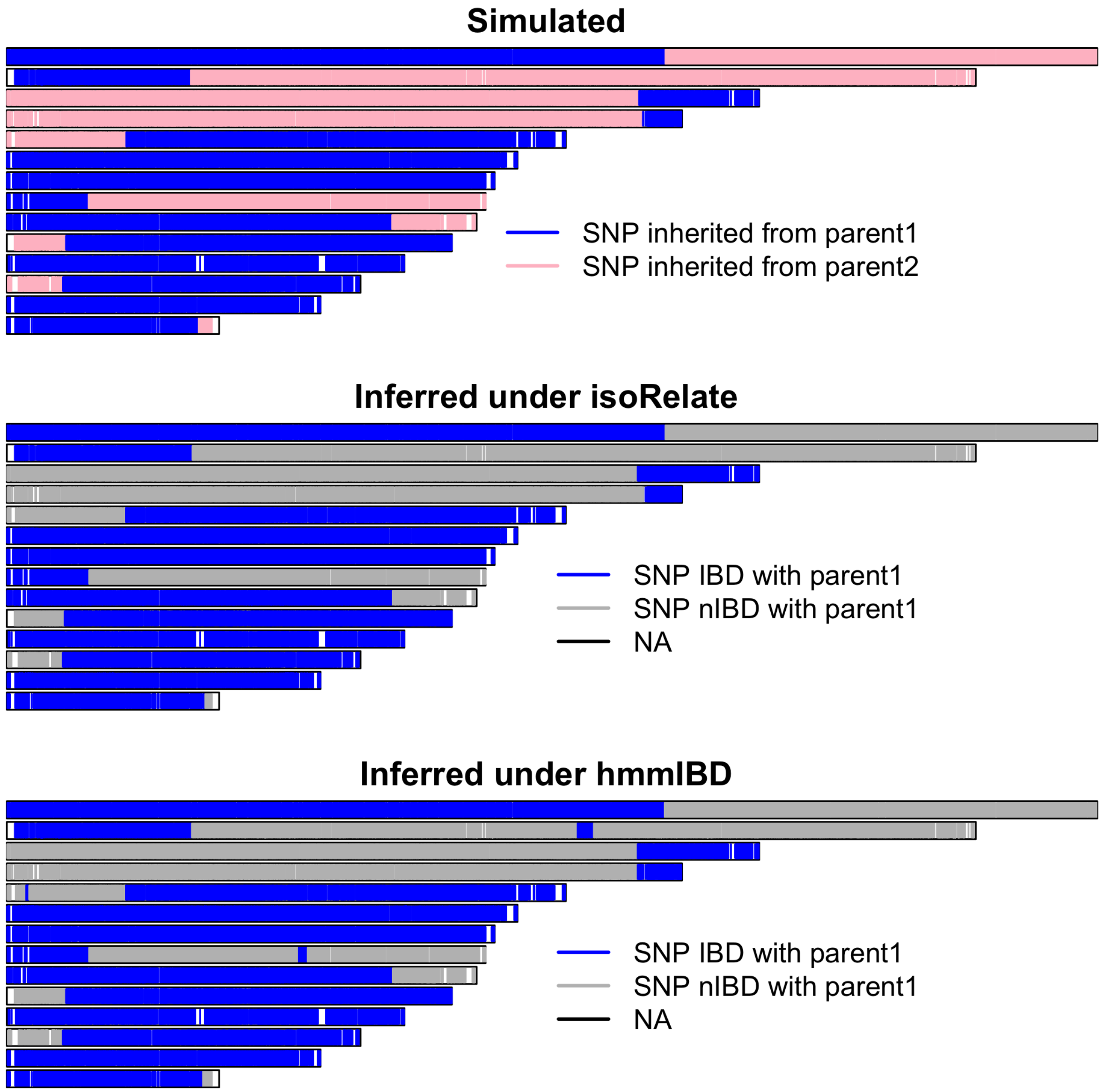
Simulated
SNP inherited from parent1
SNP inherited from parent2
Inferred under isoRelate
SNP IBD with parent1
SNP nIBD with parent1
NA
Inferred under hmmIBD
SNP IBD with parent1
SNP nIBD with parent1
NA

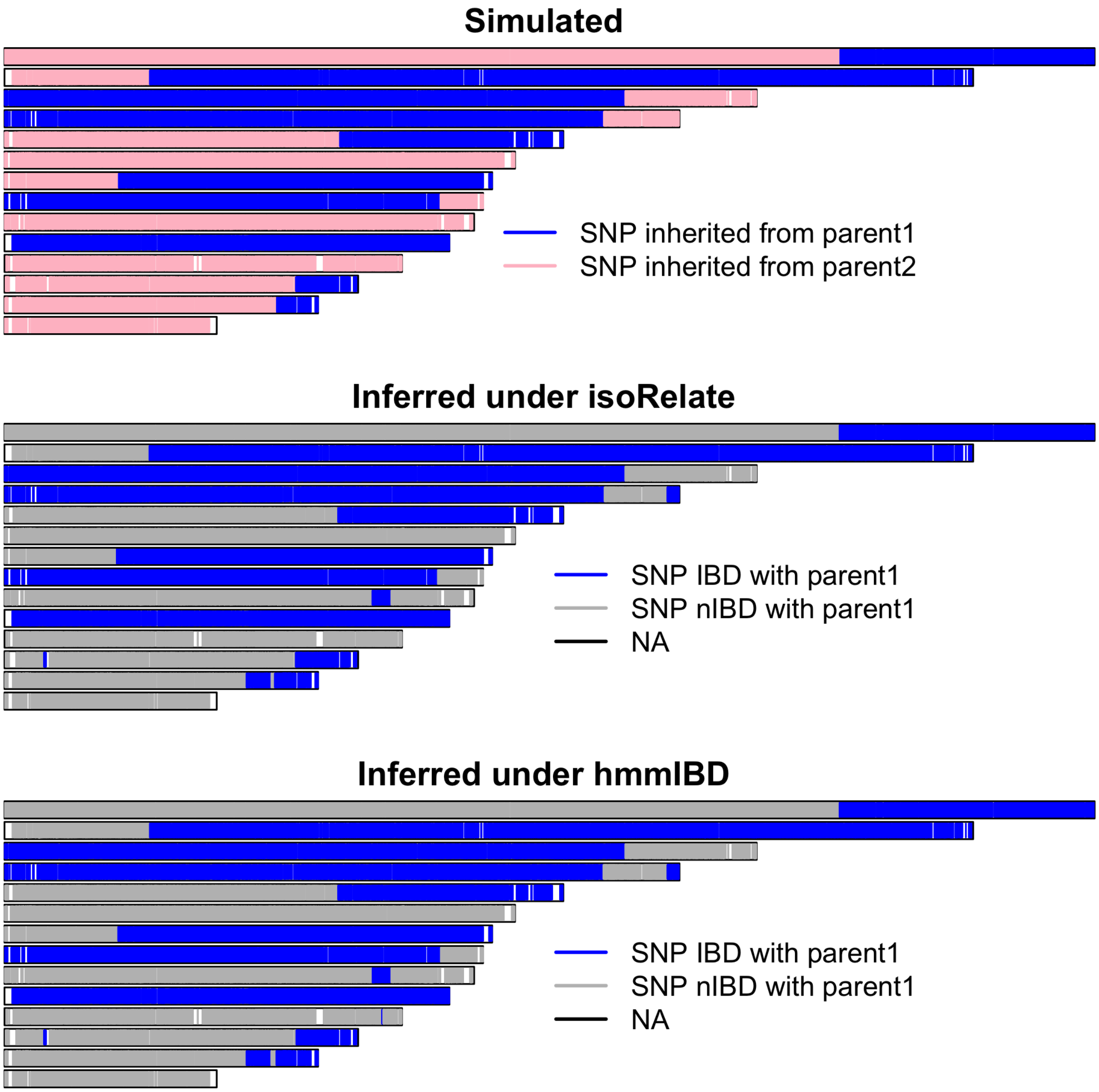

Numbers of generations based on non-erroneous chimeric children

Both methods overestimate the number of generations.

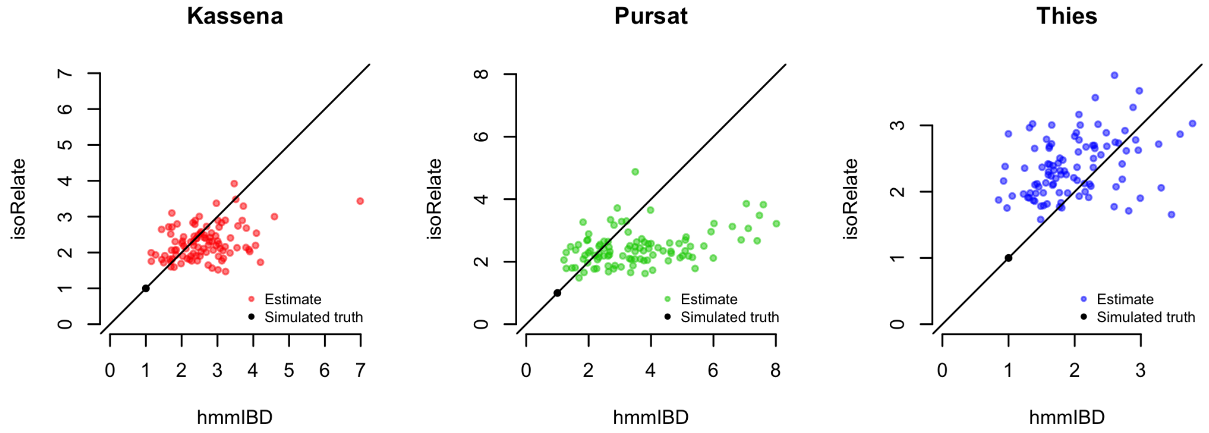

Posterior probabilities of the IBD state versus proportion simulated IBD based on non-erroneous chimeric children.

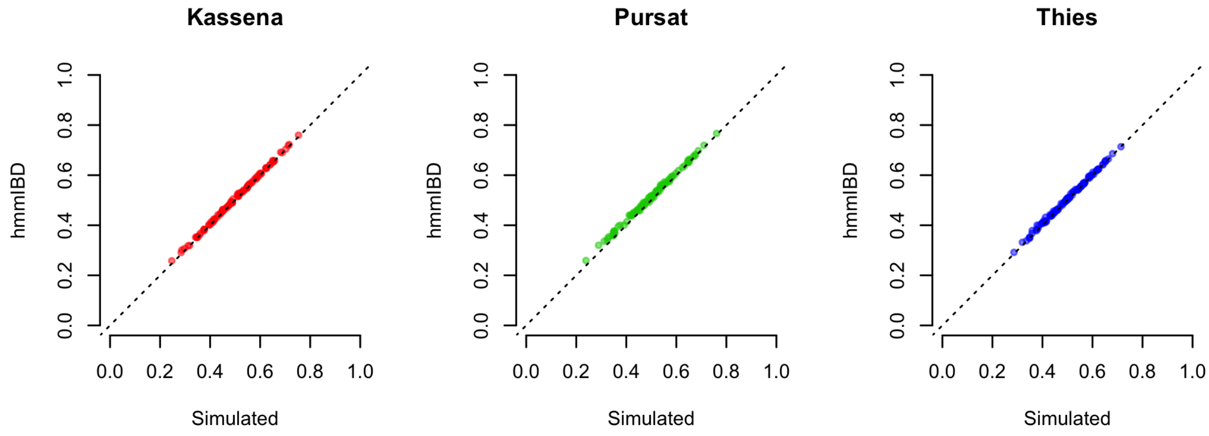
Kassena
Pursat
Thies
hmmIBD
Simulated
0.0
0.2
0.4
0.6
0.8
1.0

Inference results based on erroneous chimeric children

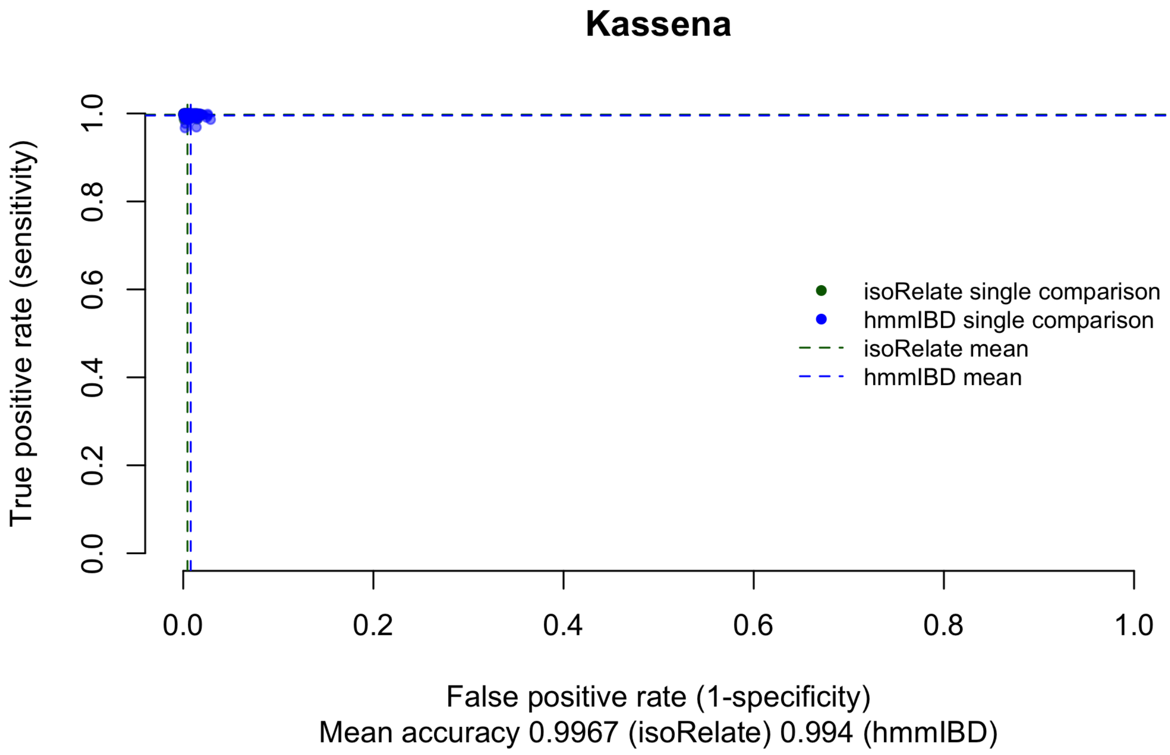

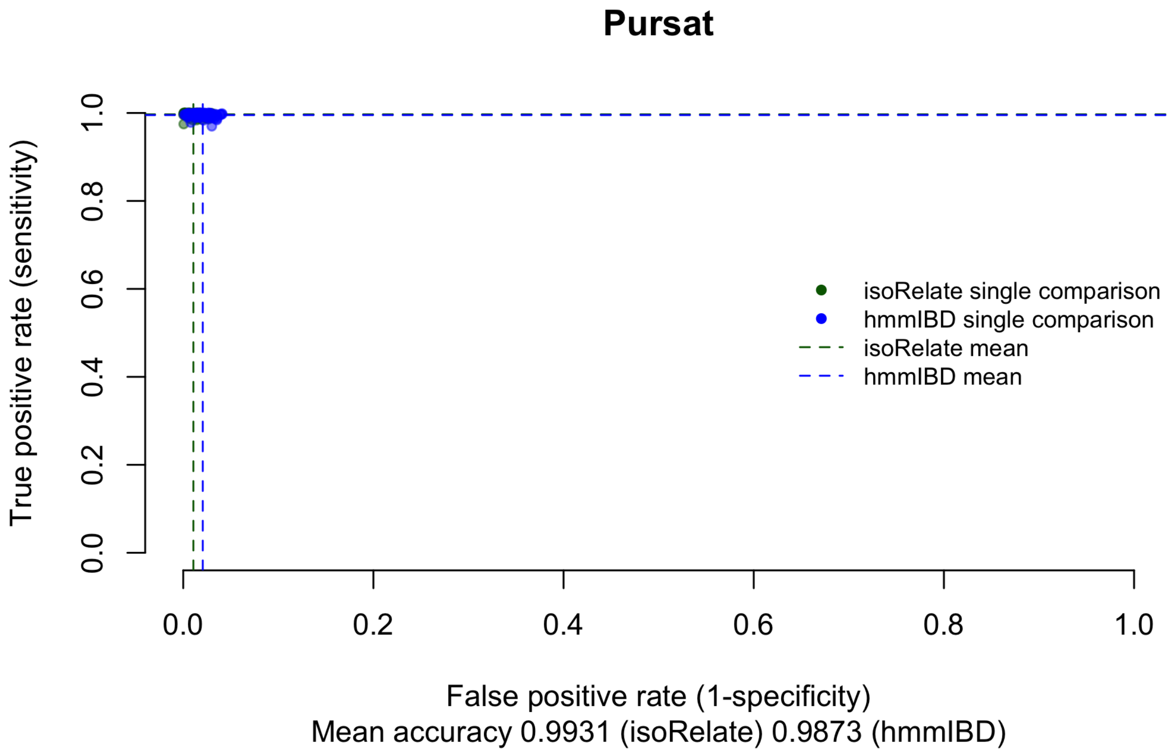

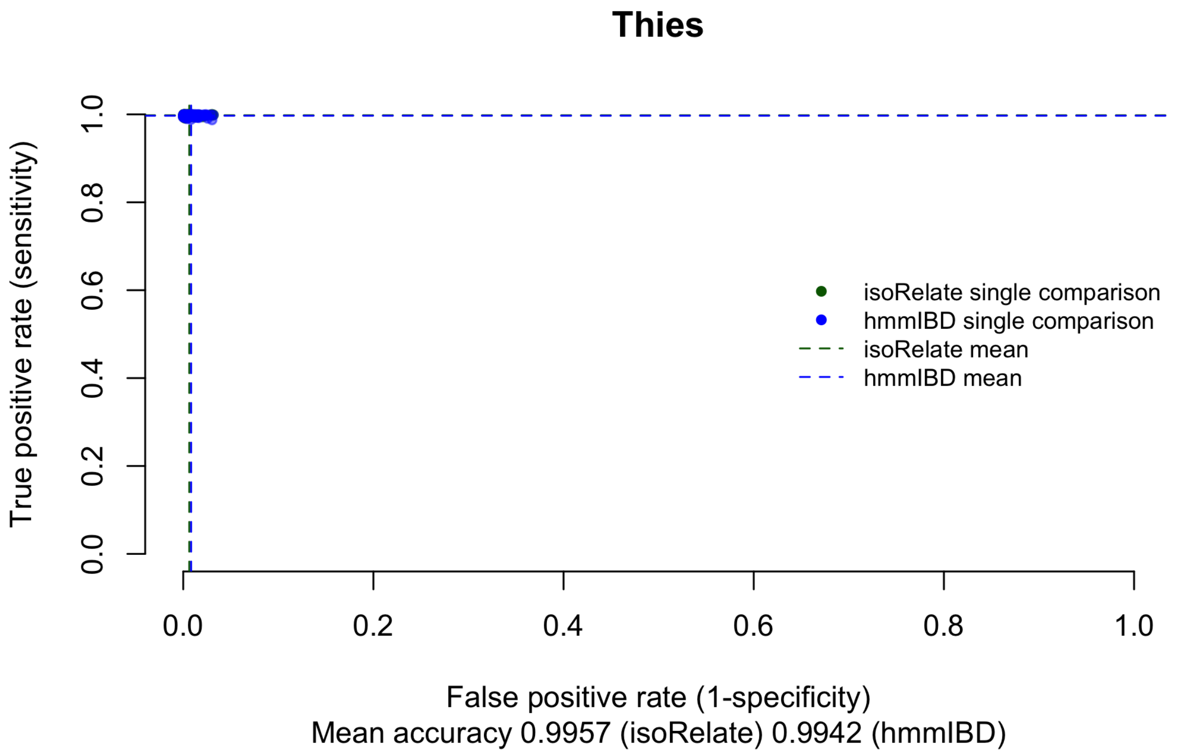

Illustrative assignment plots for two randomly selected pairwise comparisons based on erroneous chimeric children from Pursat.

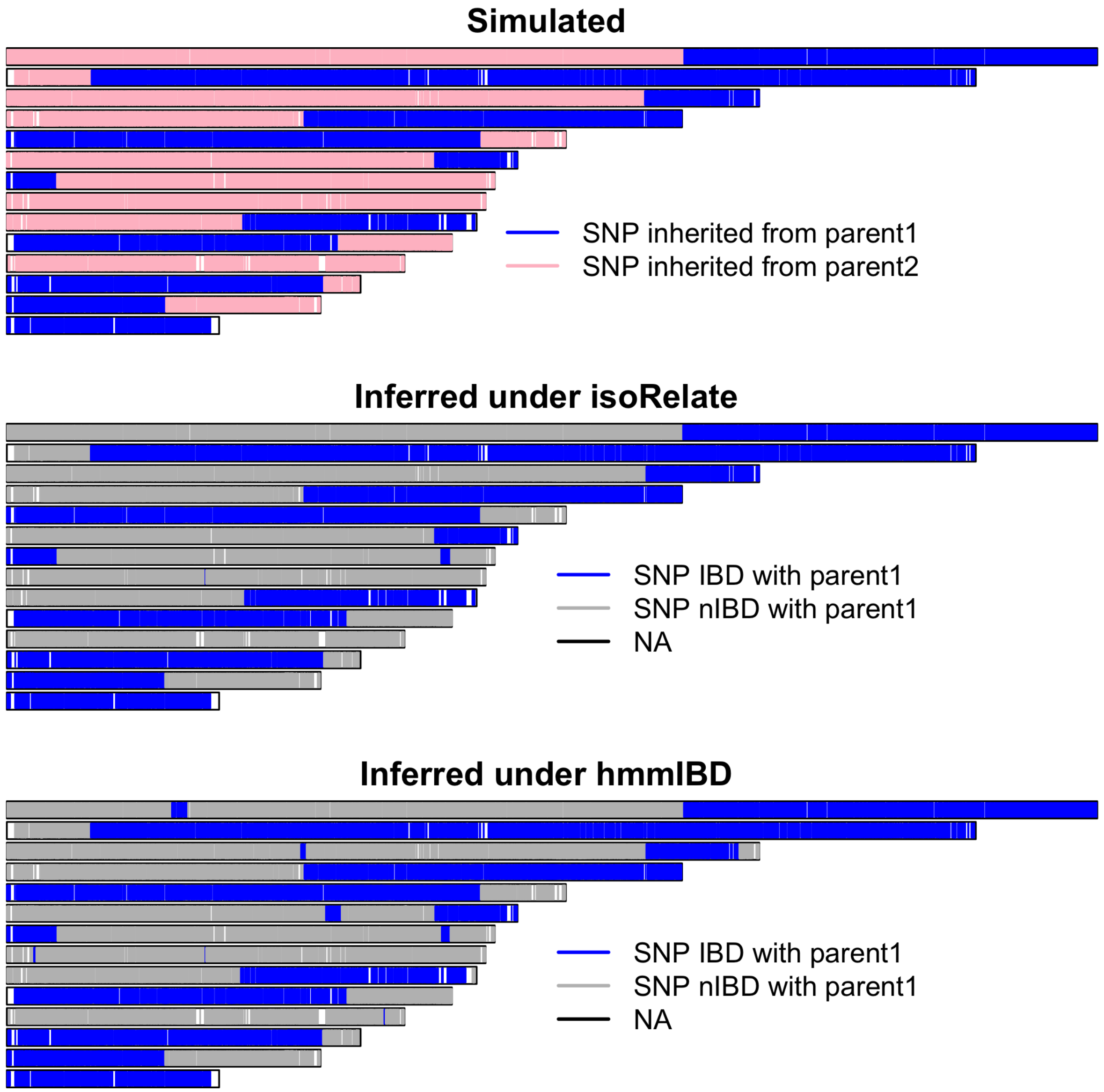
Simulated
SNP inherited from parent1
SNP inherited from parent2
Inferred under isoRelate
SNP IBD with parent1
SNP nIBD with parent1
NA
Inferred under hmmIBD
SNP IBD with parent1
SNP nIBD with parent1
NA

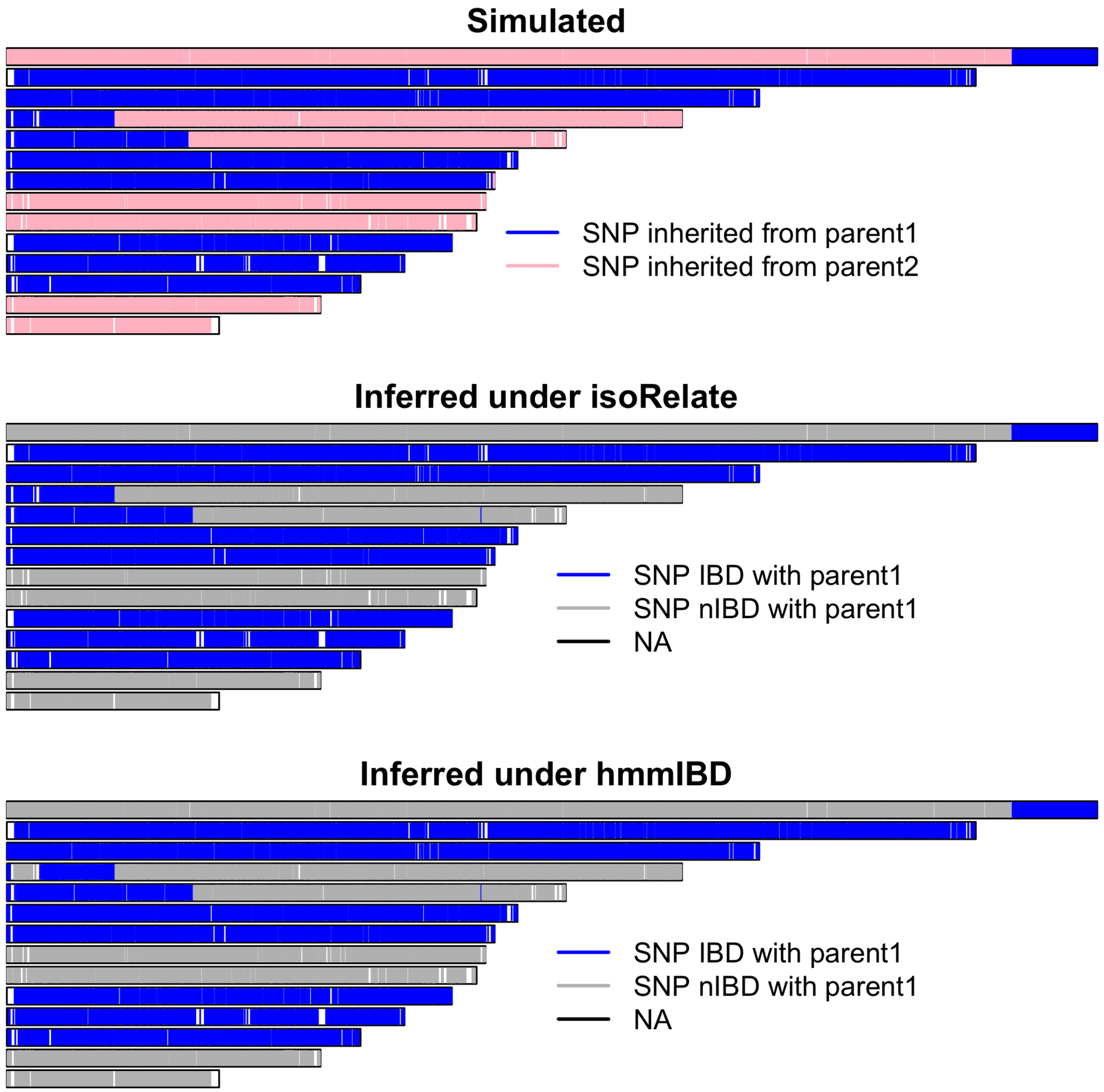

Numbers of generations based on erroneous chimeric children.

Both methods overestimate the number of generations.

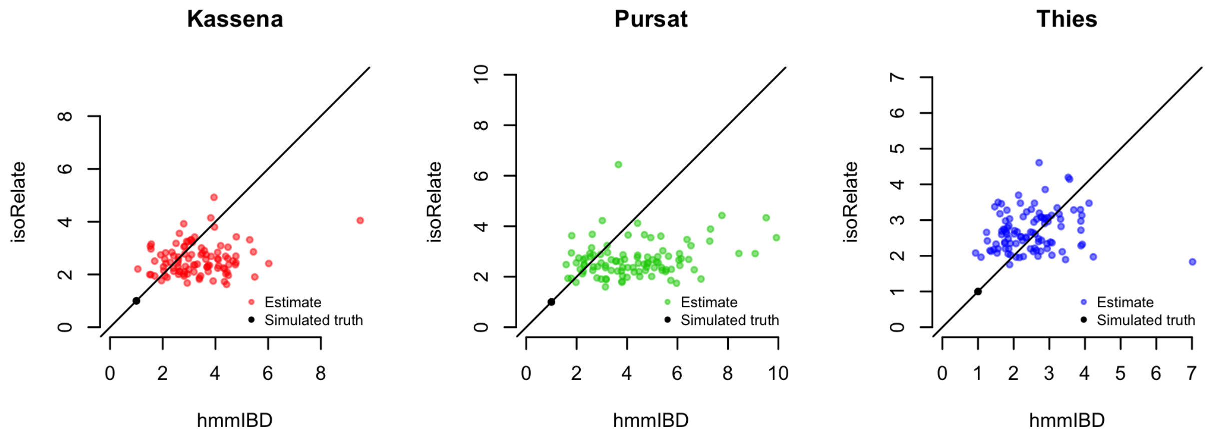

Posterior probabilities of the IBD state versus proportion simulated IBD based on erroneous chimeric children.

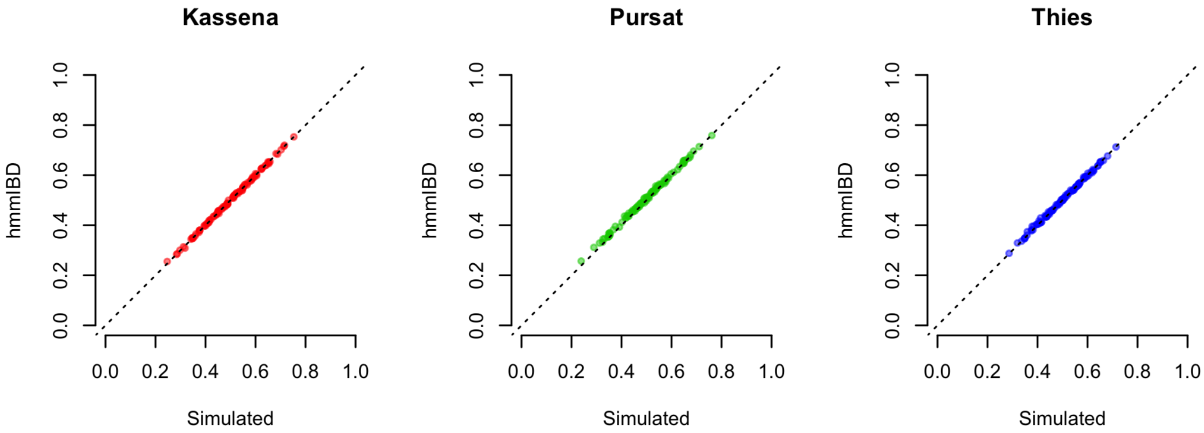

Summary

Both isoRelate and hmmIBD are highly accurate, sensitive and specific, including when genotyping error equal to 0.005 in the data is misspecified under the model at 0.001. In addition to IBD segments, hmmIBD returns the posterior IBD proportion (a measure of relatedness that integrates over all possible IBD segment assignments). Under v0.1.0 of isoRelate, posterior probabilities of the IBD state are not readily accessible, but many auxiliary functions for visualizing model output and assessing significance are provided. On average, hmmIBD was 25 times faster in user CPU time than isoRelate, but both perform adequately in real time.

Table 4: Summary of average run times for 50 samples on a MacBook Air with 1.7 GHz Intel Core i7 processor. Standard deviations in parentheses.

	Clock time (sec)	CPU time (sec)
isoRelate	1641.996 (343.287)	1583.174 (330.901)
hmmIBD	62.886 (13.309)	62.511 (13.172)

Table 5: Summary of average scores based on non-erroneous data with standard deviations in parentheses.

	Accuracy	Sensitivity	Specificity
isoRelate	0.995 (0.005)	0.999 (0.002)	0.991 (0.008)
hmmIBD	0.992 (0.006)	0.999 (0.001)	0.986 (0.011)

Table 6: Summary of average scores based on erroneous data with standard deviations in parentheses.

	Accuracy	Sensitivity	Specificity
isoRelate	0.995 (0.004)	0.997 (0.003)	0.993 (0.007)
hmmIBD	0.992 (0.006)	0.996 (0.005)	0.988 (0.01)

Impact of assuming uniform recombination under hmmIBD

To explore the impact of a misspecified uniform recombination rate under hmmIBD, we analysed data generated using a non-uniform recombination rate under hmmIBD (v2.0.0) with the default uniform recombination rate of 7.4-7 M/bp (based on the average reported in Miles A, et al. Genome Research. 2016). The data were generated alongside the data used in the comparative study as described above with the following exception. Recombination was simulated by sampling crossover events per base pair position, x, from a Bernoulli distribution with probability equal to recombination rate, $\rho(x)$, based on the following piecewise constant function (see functions.R),

$$\rho(x) = \begin{cases} 3 \times 10^{-7}\text{M/bp if } x \text{ is within 30 kb of the start or end position of the centromere} \\ 11.5 \times 10^{-7}\text{M/bp if } x \text{ is within 80-120 kb of the start or end position of the centromere} \\ 7.4 \times 10^{-7}\text{M/bp otherwise.} \end{cases} \quad (4)$$

where the start and end positions of the centromeres are based on Table S2 of Miles A, et al. Genome Research. 2016. Equation (4) is based on findings reported in Miles A, et al. Genome Research. 2016. Specifically, Miles et al. found that within approximately 30 kb of the centromere, the recombination rate was significantly lower than average; and that between approximately 80 and 120 kb of the centromere, the rate was slightly higher than average. Based on Figure 3C of Miles A, et al. Genome Research. 2016., the lower rate is approximately equal to 0.3 M/Mbp (3×10^{-7} M/bp), the slightly higher rate is approximately equal to 1.15 M/Mbp (11.5×10^{-7} M/bp), while the average rate is equal to 0.74 M/Mbp (7.4×10^{-7} M/bp).

As above, accuracy, sensitivity and specificity were high (Table 7 and Figures 1 and 2); hmmIBD overestimated the number of generations (Figure 3), but posterior probabilities closely matched the proportion simulated IBD (Figure 4).

In summary, given deviations within a biologically informed range (Miles A, et al. Genome Research. 2016.), the assumption of uniform recombination has little impact upon inference on IBD under hmmIBD using data within the accessible genome. The assumption is unlikely to hold over regions where the recombination rate deviates greatly from the average over the accessible-genome, however. We therefore recommend exclusion of such regions (e.g. subtelomeric regions) in data analysed under hmmIBD.

Table 7: Summary of average scores based on data generated using a non-uniform recombination rate with standard deviations in parentheses.

	Accuracy	Sensitivity	Specificity
Kassena	0.993 (0.004)	0.999 (0.001)	0.987 (0.007)
Pursat	0.986 (0.007)	0.999 (0.002)	0.973 (0.012)
Thies	0.993 (0.004)	0.998 (0.002)	0.988 (0.008)

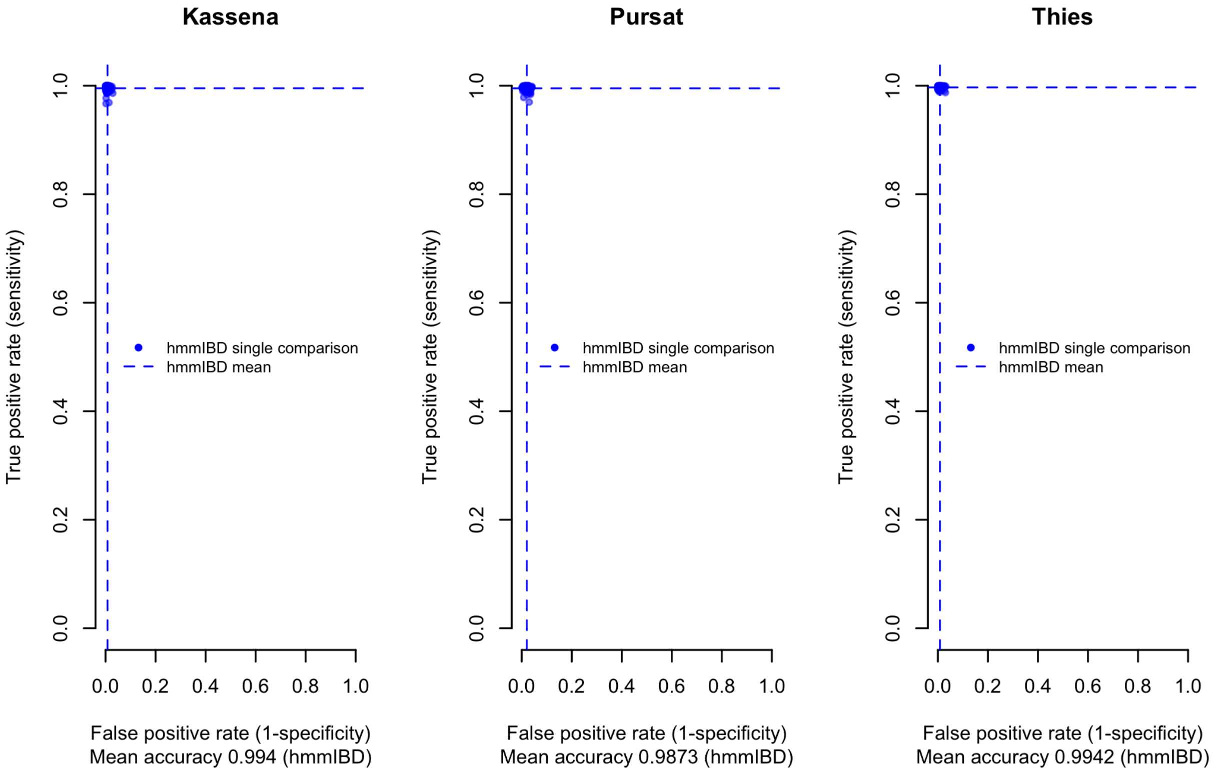

Figure 1: Plots of scores based on data generated using a non-uniform recombination rate.

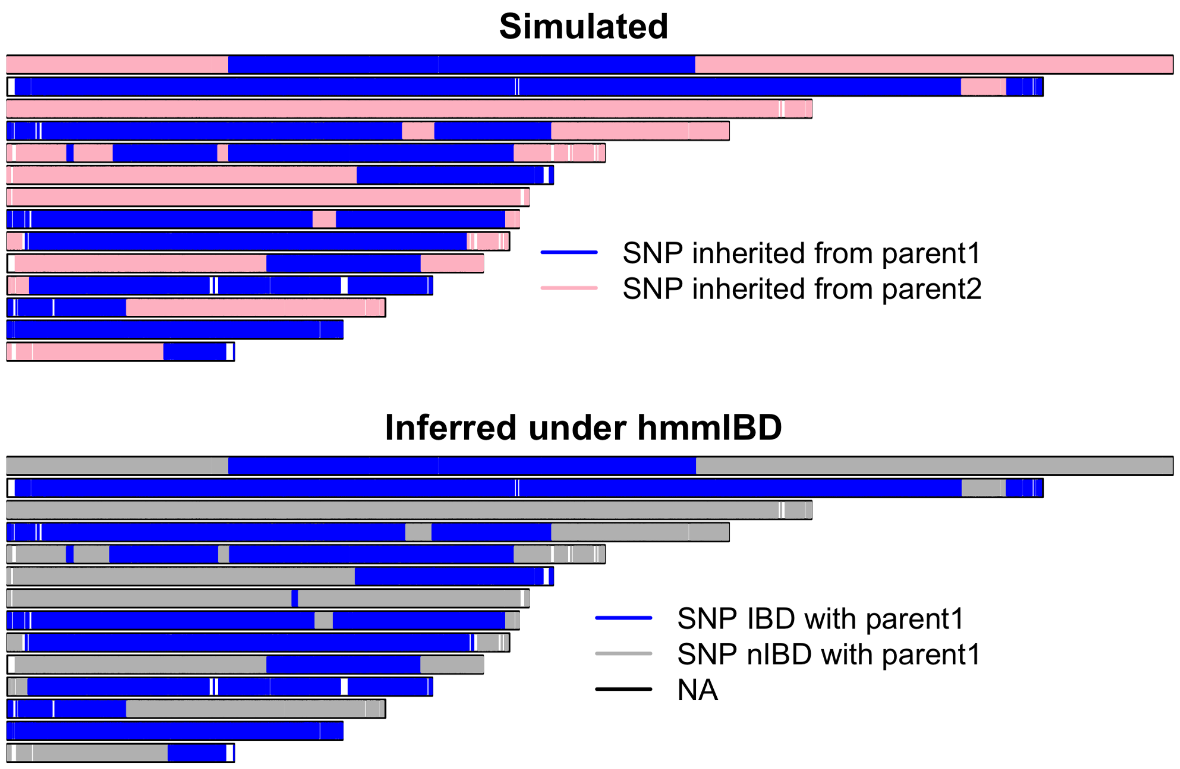

Figure 2: Illustrative assignment plots for a randomly selected pairwise comparison based on chimeric child from Thies generated using a non-uniform recombination rate.

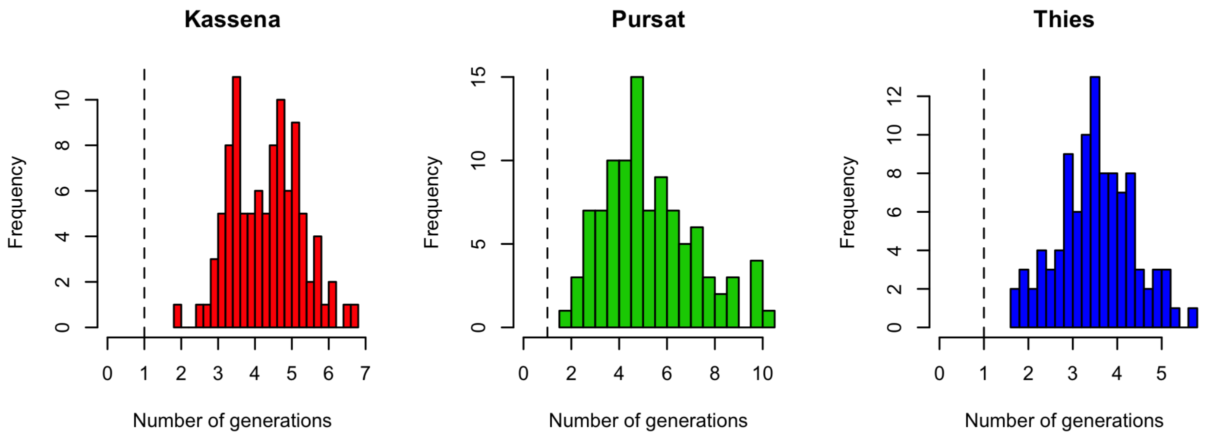

Figure 3: Histograms of numbers of generations estimated from data generated using a non-uniform recombination rate. The true number of generations equal to one is denoted by a dashed vertical line.

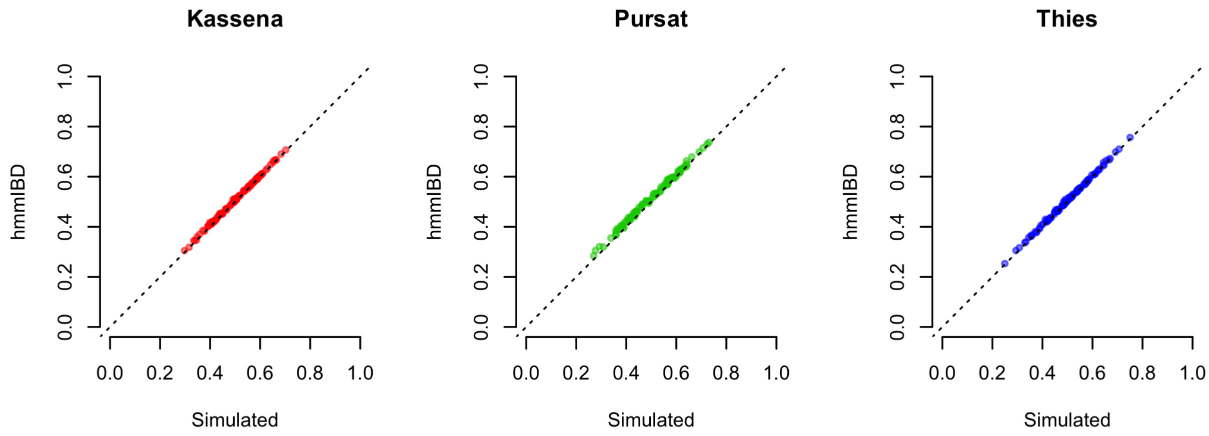

Figure 4: Posterior probabilities of the IBD state inferred from data generated using a non-uniform recombination rate.
